# Inferring Host Gene Subnetworks Involved in Viral Replication

**DOI:** 10.1371/journal.pcbi.1003626

**Published:** 2014-05-29

**Authors:** Deborah Chasman, Brandi Gancarz, Linhui Hao, Michael Ferris, Paul Ahlquist, Mark Craven

**Affiliations:** 1 Department of Computer Sciences, University of Wisconsin–Madison, Madison, Wisconsin, United States of America; 2 Department of Biostatistics and Medical Informatics, University of Wisconsin–Madison, Madison, Wisconsin, United States of America; 3 Luminex Corporation, Madison, Wisconsin, United States of America; 4 Institute for Molecular Virology, University of Wisconsin–Madison, Madison, Wisconsin, United States of America; 5 Howard Hughes Medical Institute, University of Wisconsin–Madison, Madison, Wisconsin, United States of America; 6 Morgridge Institute for Research, University of Wisconsin–Madison, Madison, Wisconsin, United States of America; National Center for Biotechnology Information (NCBI), United States of America

## Abstract

Systematic, genome-wide loss-of-function experiments can be used to identify host factors that directly or indirectly facilitate or inhibit the replication of a virus in a host cell. We present an approach that combines an integer linear program and a diffusion kernel method to infer the pathways through which those host factors modulate viral replication. The inputs to the method are a set of viral phenotypes observed in single-host-gene mutants and a background network consisting of a variety of host intracellular interactions. The output is an ensemble of subnetworks that provides a consistent explanation for the measured phenotypes, predicts which unassayed host factors modulate the virus, and predicts which host factors are the most direct interfaces with the virus. We infer host-virus interaction subnetworks using data from experiments screening the yeast genome for genes modulating the replication of two RNA viruses. Because a gold-standard network is unavailable, we assess the predicted subnetworks using both computational and qualitative analyses. We conduct a cross-validation experiment in which we predict whether held-aside test genes have an effect on viral replication. Our approach is able to make high-confidence predictions more accurately than several baselines, and about as well as the best baseline, which does not infer mechanistic pathways. We also examine two kinds of predictions made by our method: which host factors are nearest to a direct interaction with a viral component, and which unassayed host genes are likely to be involved in viral replication. Multiple predictions are supported by recent independent experimental data, or are components or functional partners of confirmed relevant complexes or pathways. Integer program code, background network data, and inferred host-virus subnetworks are available at http://www.biostat.wisc.edu/~craven/chasman_host_virus/.

## Author Summary

Nearly every step of the viral life cycle requires the action or use of host machinery. Genome-wide suppression experiments have been used to identify individual host genes whose products are involved in viral replication. The hit sets identified by such experiments are typically fairly large and difficult to comprehend. We propose a method to infer subnetworks of intracellular interactions that explain the experimental data. These inferred subnetworks make the data more interpretable in terms of the mechanisms of viral replication and can be used to guide further experiments.

## Introduction

A virus requires host cellular machinery to complete its life cycle. Understanding the interactions that occur between viruses and their hosts can contribute to the development of preventative and therapeutic methods to control their effects on human health. To this end, an increasing number of genome-wide loss-of-function studies have recently been performed to identify host factors that modulate the virus life cycle in a host cell. These studies have used either yeast mutant libraries [1]–[5] or RNA interference [6]–[10] to systematically suppress the production of host gene products. For each host gene that is manipulated, the effect on the virus is assessed by measuring the replicative yield of viral genetic material or viral proteins relative to a control. Typically, these genome-wide screens identify a large number of host genes, which we refer to as *hits*, whose loss has a significant effect on the virus. However, the screens themselves do not reveal how the gene products of these hits are organized into the pathways that modulate the virus, nor do they indicate which host gene products directly interface with a viral component. We consider the computational task of inferring directed subnetworks that hypothesize the pathways through which each hit modulates viral replication. The value of these inferred subnetworks is that they can be used to (i) predict which unassayed genes may be involved in viral replication, (ii) interpret the role of each hit in modulating the virus, and (iii) guide further experimentation that is aimed at uncovering and validating the mechanisms of host-virus interaction.

We present an approach that uses an integer linear program (IP, for brevity) to infer the pathways that are involved in the lifecycle of a virus in a host cell. The inputs to our approach are the list of phenotypes measured in a genome-wide loss-of-function assay, including a list of those host genes that are hits, and a partially-directed *background network* characterizing known physical interactions among host cellular components. Using these data, our approach predicts the identity of a small number of host-virus *interfaces* (host factors that are closest to a direct interaction with the virus), and infers a subnetwork of directed interactions that provides at least one path from every hit to a predicted interface. By providing these paths, we say that the subnetwork plausibly *explains* or *accounts for* the viral phenotype observed when each hit is suppressed. Because the background network and experimental observations are incomplete, many different subnetworks may be inferred for the same set of hits. To account for this, our method infers an ensemble of subnetworks, each of which provides paths for all of the hits. We use the ensemble to assess our confidence in various aspects of the predicted subnetworks.

**Figure 1:**
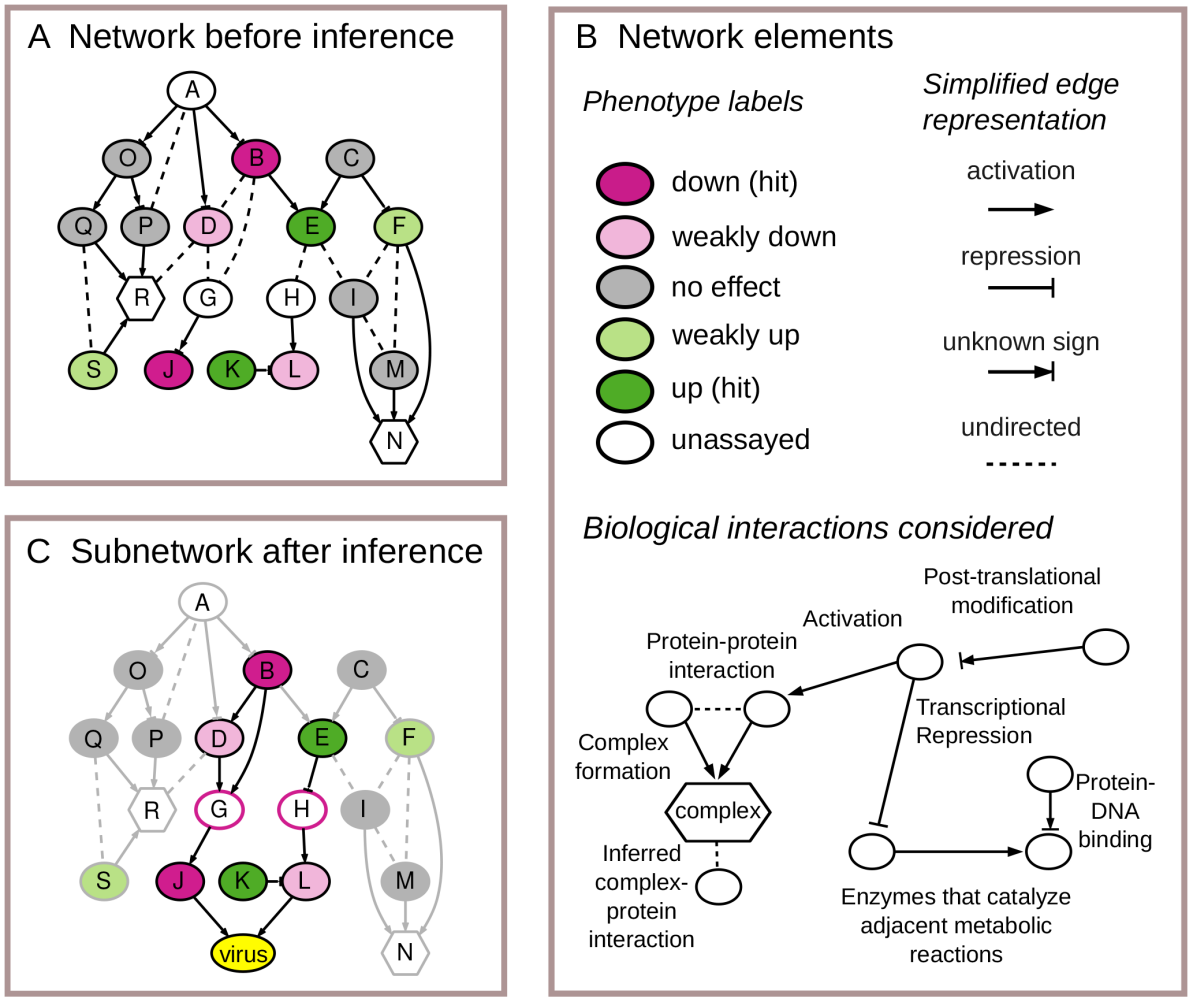
Input and output for our subnetwork inference approach. (**A**) The inputs to our subnetwork inference approach are phenotypes measured in a loss-of-function assay and a background network characterizing known interactions. (**B**) The network elements represented in panels **A**, **C**, and other figures. (**C**) An inferred subnetwork for the given inputs. The subnetwork includes a directed, consistent path linking each hit (gene with an **up** or **down** phenotype) to the virus. The red borders on the unassayed nodes G and H indicate that they are inferred to have the **down** phenotype. Edges shown in gray are not included in the subnetwork.

Figure 1 provides an illustration of the input and output of our computational approach. Figure 1(A) shows what is provided as input to the approach using a graph representation. Nodes in the graph represent host genes, proteins, and protein complexes. Both the gene and its encoded protein are represented using the same node. The connecting edges in the graph provide a simplified representation of known interactions among the nodes.

Figure 1(B) presents a graphical guide to the network elements used by our method. The color of a gene node specifies the observed phenotype when expression of the gene's product is suppressed. Using the loss-of-function assay data, we derive discrete viral phenotype labels that describe the sign and magnitude of the measured effect of each host gene on viral replication: **down** and **weakly down** for genes whose loss of function reduces viral replication, **up** and **weakly up** for genes whose loss increases viral replication, and **no-effect** for genes with no consistent, measurable effect on viral replication. The figure also shows the types of interactions in the background network and how they are distilled into a simplified representation. Each interaction is represented by an edge indicating the *direction* and *sign* (activation or inhibition) of the interaction, when these properties are known.

Figure 1(C) shows the result of the inference process, which is a directed subnetwork that accounts for the loss-of-function phenotype of each hit (B,E,J,K) by providing potential mechanistic paths leading to a direct interaction with the virus. In the subnetwork shown, host gene products J and L are predicted to be interfaces between the host and virus, as indicated by the directed edges to the virus node. Some of the edges and nodes, shown in gray, are deemed to be not relevant to viral replication, and hence not useful for explaining the measured hits; these include all genes with **no-effect** (gray) viral phenotypes. The dark edges, which are considered part of the inferred subnetwork, are assigned directions and signs in cases where these properties are not specified by the background network. The directions for the relevant edges are set so that for each hit, there is at least one path that proceeds forward from it to the virus. The signs for the relevant edges are set so that each one gives a biologically plausible interpretation of how the interaction is relevant to viral replication. For example, protein E has an **up** phenotype and modulates the virus by inhibiting the expression or function of protein H, which activates the function or expression of the interface protein L. Additionally, the subnetwork predicts that two genes whose phenotypes are unknown (G, H), and two genes whose phenotypes are weak (D, L), are actually key host factors involved in viral replication.

The integer linear program used in our approach consists of an objective function and a set of constraints characterizing subnetworks that are deemed biologically interpretable. Due to functional redundancy in the host genome and the inability to assay some host-gene suppressions, many true hits are not identified by individual loss-of-function experiments. Therefore, to predict additional hits and to identify multiple paths between hits and interfaces, our objective function maximizes the inclusion of unassayed genes and genes with weak viral phenotypes, subject to other constraints on the subnetwork. These genes are prioritized using a diffusion kernel (DK) scoring method, which assigns scores to genes based on their network proximity and connectivity to the hits. As a counterpoint to the objective function, which is generous in including genes in the subnetwork, the IP's constraints provide restrictions on which paths may be inferred to be part of the subnetwork. All of the inferred paths must be *directed*, meaning that each interaction in a path is directed forward from the hit to the virus, and directions are inferred for undirected interactions. The paths must also be *consistent*, meaning that the sign (activating or inhibitory) of each interaction between host factors agrees with the viral phenotypes of the interactors. For a pair of host factors that both inhibit or both facilitate viral replication when suppressed, an activating interaction is consistent. For a pair of host factors that affect the virus in opposite ways, an inhibitory interaction is consistent. Using these rules, our method infers the signs of unsigned interactions and the viral phenotypes for unassayed host factors.

We assess the inferred subnetworks using both computational experiments and an analysis of the relevant literature. First, we conduct a cross-validation experiment to evaluate the accuracy of our inferred subnetworks in predicting host factors involved in viral replication. We compare the accuracy of our approach to several baselines including a diffusion kernel method which is used as an input to our approach. Our results demonstrate that (i) the high-confidence predictions of our IP approach achieve a high level of accuracy, (ii) the predictions made by our method are more accurate than those made by several baselines, and (iii) the accuracy of our method for this task is comparable to the diffusion kernel method which does not infer detailed causal pathways like our IP approach. Second, we use our approach to predict a set of host-virus interfaces and a set of unassayed host genes that are likely to be modulators of viral replication. We discuss independent biological evidence that supports a number of these predictions. Finally, we perform a suite of additional computational experiments to assess our method's predictions in other ways. These include (i) a comparative analysis to IP components inspired by related work, (ii) a Gene Ontology analysis to evaluate the ability of our inferred subnetworks to better identify relevant functional categories than an analysis of the experimental data alone, and (iii) a Monte Carlo analysis to assess whether the protein complexes that our method predicts to be relevant are well supported by the experimental data and subnetwork-inference process.

### Related work

Our work is related to methods that address several different categories of problems: finding mechanistic explanations for source-target pairs, subnetwork extraction, candidate gene prioritization, and gene set enrichment.

One closely related task is to infer the physical interactions that mediate the observed direct or indirect relationships between a source gene and a target gene. The input to these methods is a set of source-target pairs and a background network consisting of unsigned protein-protein and/or protein-DNA interactions. The output is a subnetwork that provides a connection between each source and target. Most closely related to our work are the approaches that globally infer a subnetwork to account for all given pairs by providing paths between them. The Markov network-based Physical Network Model [11] and the integer programming-based SPINE [12] both infer subnetworks in which each source must be connected to its targets by one or more acyclic pathways, and in which the sign of each edge is also inferred. The Physical Network Model also infers directions for edges. Related methods for signaling network orientation [13]–[15] infer edge directions, but not edge signs or node phenotypes. Yosef *et al*. [16] infer rooted trees that connect a set of sources with a set of targets. Additionally, some methods account for source-target pairs separately, rather than in a global inferred subnetwork [17], [18]. Others employ genetic interactions or correlation of mRNA expression in addition to protein-protein interactions to infer indirect or direct relationships between genes [19], [20]. Our work has similarities to these approaches, particularly those based on integer linear programming, but differs in some key respects. In our setting, the common target of all hits – the virus – is external to the background network, and the identity of the host factors that interact with it directly must be predicted. Additionally, our background network encompasses a greater variety of biological interactions than the background networks used by these other approaches. Unlike the methods that use mRNA expression profiles as the basis for determining direct or indirect relationships between genes, ours uses only phenotypes derived from a genome-wide mutant assay.

Recently, Gitter et al. [21] presented an application of their source-target pair-based method to inferring human signaling pathways involved in influenza A viral infection. In their approach, sources are human proteins that are known to directly interact with a viral component, analogous to the interfaces in our conceptual model. Targets are human genes whose expression is measured over several time points during viral infection. The method orients paths through a protein-protein interaction network from the sources to the targets, preferring paths that contain influenza-relevant genes identified by RNAi experiments. Conceptually, this method infers the signaling pathways that control the host's transcriptional response to viral infection. In this paper, we look at host-virus interactions from the opposite direction and infer the mechanistic pathways by which suppressed host genes inhibit or enable the normal viral replication cycle.

Other related methods address the network extraction task: selecting specific types of connecting structures from a background network when a biologically-motivated node- and/or edge-weighting function is available. The structures include rooted trees [22], variants on Steiner trees [23]–[26], random walks and short paths [27], parallel pathways [28], dense highly-connected subnetworks [29], and undirected subnetworks that provide connections between pairs of genes [30]. Unlike our method, these approaches do not distinguish (or infer) phenotype signs and edge signs, nor do they apply global constraints to the extracted subnetwork other than a global edge minimization. In contrast, we employ global constraints such as an upper bound on the number of interfaces. We do not believe that for our task it is appropriate to assume the entire network will be minimal, which is an assumption made by the Steiner tree and shortest-paths methods.

Other methods apply graph kernels or flow algorithms to an interaction network to predict and prioritize additional hit genes [31]–[34]. Notably, Murali *et al*. [33] predict which genes modulate HIV replication in human cell lines. Like these methods, our approach uses a gene ranking method to prioritize genes for inclusion in the inferred subnetwork. However, these methods themselves do not infer consistent, directed pathways, nor do they predict which host factors directly interact with the virus. Our approach combines a gene prioritization method with a directed network inference method.

More distantly related to our work, gene set enrichment techniques are widely used to interpret hit sets identified by high-throughput experiments. These methods identify which pre-defined biological components and processes, such as Gene Ontology annotations or KEGG pathways [35], [36], are represented in a set of genes [37]. In contrast, our method does not restrict our pool of candidate genes and interactions to predefined gene sets. Additionally, gene set enrichment-based methods are typically better suited when the task is to identify common annotations within a gene set, rather than to predict a set of high-precision additional hits or relevant mechanistic interactions among known hits.

## Materials and Methods

### Data

The input to our approach consists of a set of viral phenotypes observed in a loss-of-function experiment and a background network of intracellular interactions. When available, we can also take advantage of confirmed relevant interactions curated from the literature.

#### Experimental observations

We analyze data from experiments screening the yeast genome for genes that modulate the replication of two RNA viruses: Brome Mosaic Virus (BMV) [1], [4] and Flock House Virus (FHV) [5]. The experiments measure the replication of the virus in a yeast host when the expression of one gene is partially or completely depleted. Yeast mutant strains allow the majority of cell genes (of about 5,800 total genes in yeast) to be screened in parallel. For nonessential genes, the experiment was performed using the yeast deletion library [38]. Essential genes were screened using a collection of yeast strains, each with a single essential gene promoter replaced by a doxycycline-repressible promoter, allowing repression of gene expression by adding doxycycline to the growth medium [39]. Each data set includes at least two replicate assays for each mutant strain.

As yeast is not the natural host for either virus, an artificial experimental system was used to initiate viral replication. Each mutant yeast strain was grown and transformed with two DNA plasmids expressing viral components. The plasmid expressing viral RNA also contained a luciferase reporter gene, allowing the accumulation of viral RNA to be measured by the intensity of the light produced from luciferase gene expression. The output of the assay is the *fold-change* in accumulation of viral RNA between each mutant strain and the control. Let 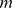 be the virus expression level in the mutant strain, and 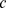 be the expression level in the control strain. Fold-change is computed as 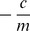 if 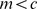, or 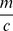 if 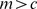.

We derive a discrete phenotype label for each assayed gene based on the sign, magnitude, and reproducibility of the fold-change across replicate assays. If a mutant reproducibly yields a decrease in viral replication, the interpretation is that the missing gene product directly or indirectly facilitates virus replication. We label such mutants with a **down** or **weakly-down** phenotype, depending on the magnitude of the fold-change. Conversely, the interpretation for a mutant that reproducibly results in an increase in viral replication is that, when expressed, the missing gene product directly or indirectly inhibits the replication of the virus. We label such mutants **up** or **weakly-up**. The mutants with high-magnitude phenotypes, **down** and **up**, are considered *hits*. While we include mutants with weak phenotypes in our analysis, we are primarily interested in explaining the hits.

The threshold used to divide the hit and weak phenotypes was determined separately for each screen and, for BMV, is described in greater detail in the original publications. In the BMV data set, different thresholds were used for essential and nonessential genes. To be considered a hit for BMV, a nonessential gene mutant resulted in at least a 2.5-fold change in two replicates and at least an average 3-fold change. A more stringent threshold was used for essential gene mutants, which cause expression knockdown rather than complete knockout. Essential gene hits conferred at least a 6-fold change in BMV expression in two replicates. As for FHV, the data set consists of only nonessential yeast gene mutants. FHV hits conferred at least a 2-fold change in viral replication in two replicates, and additionally passed a secondary validation by northern blot.

We assign a third category of phenotype, **no-effect**, to genes for which the sign of the fold-change is different across replicates. Finally, genes that were either not screened, or for which the yeast colony did not grow, are labeled **unobserved**. Table 1 presents the distribution of phenotypes considered here for the BMV and FHV assays. While all available gene mutants were assayed in the experiments, we limit our analysis to only those genes that are represented in the background network.

**Table 1:** Phenotype labels for suppressed host genes.

Phenotype	BMV	FHV
**up** (hit)	49	48
**weak-up**	623	826
**weak-down**	1,067	668
**down** (hit)	55	7
**no-effect**	1,074	991

Distribution of phenotype labels for genes in the background network. The labels were derived from genome-wide assays of Brome Mosaic Virus and Flock House Virus replication in yeast.

#### Background network

The interactions in the subnetworks inferred by our method are drawn from a background network that we have assembled from various publicly available data sets. The entities represent gene products and protein complexes. The interactions describe protein-protein and protein-DNA interactions, post-translational modifications of proteins, protein complex membership, transcriptional regulatory interactions, metabolic pathways, and inferred physical interactions between complexes and proteins. In concordance with our goal of inferring mechanistic subnetworks, nearly all interaction types represent direct physical interactions. The exception is the metabolic pathway interactions, which are edges between enzymes that catalyze adjacent metabolic reactions.

High-confidence interactions were selected from each database using stringent filters; for example, protein-protein interactions were selected from BioGRID [40] only if the interaction was observed using at least two different types of experimental methods. In total, the background network consists of 4,667 entities and 14,447 interactions. Node and edge counts and citations for the intracellular interaction network are described in Tables 2 and 3.

**Table 2:** Types of host factors represented by nodes in the background network.

Node type	Count
Yeast ORFs	4,167
Protein complexes	472
Small RNAs	15
Mitochondrial ORFs	8

**Table 3:** Intracellular interactions in the background network.

Interaction	Source	Directed	Signed	Count
Protein-protein	[40]	N	N	4,132
Inferred complex-complex interactions	[40]–[42]	N	N	22
Inferred complex-protein interactions	[40]–[42]	N	N	1,128
Between metabolic enzymes	[41]	N	N	713
Between metabolic enzymes	[41]	Y	N	440
Post-translational modifications	[40]	Y	N	514
Protein-DNA, unsigned	[71]	Y	N	4,067
Protein-DNA, signed	[72], [73]	Y	Y	1,248
Complex membership	[41], [42]	Y	Y	2,183

Binary interactions in the background network.

Since we are focused on inferring the direction and consistency of paths, we do not need to represent all of the distinctions among the various types of interactions in our background network. Instead, we use a simple, general representation. In this representation, both genes and their gene products are represented using the same node; in this text, we identify nodes using the protein name. Each edge may have a direction and a sign. The direction determines which interactor is the source, and which is the target. For example, for a protein-DNA interaction, a transcription factor is the source, and the regulated gene is the target. The sign describes the effect, positive or negative, of the source on the synthesis, stability, or specific activity of the target. A positive sign is called *activation*, whereas a negative sign is called *inhibition*. Many edges in the background network are not provided with a sign or direction. For example, transcription factor-gene binding interactions and post-transcriptional modifications are directed but unsigned, and most protein-protein interactions are undirected and unsigned.

Most of the interaction data sets we use are already encoded as binary interactions. However, we extract binary edges from two additional data sets that were not originally in that format: metabolic pathway data and protein complex membership data. To extract binary interactions from the metabolic pathway data [41], we draw an edge between enzymes that catalyze adjacent reactions. This edge is directed unless both reactions were annotated as reversible.

We also represent protein complexes in the background network. Pu *et al*. [42] and Heavner *et al*. [41] provide manually-curated protein complex information in the form of sets of genes that are each labeled with the name of a protein complex. To represent the protein complexes, we first add a node that represents the complex, and next add activating, directed edges from each constituent gene to the complex node. Protein complex nodes are treated the same as any other node. One implication of our representation is that only the components of a complex that share the same phenotype label will be drawn into predicted relevant paths that involve the complex.

We also infer a set of undirected complex-complex and protein-complex interactions by combining the protein complex membership information [41], [42] with the protein-protein interactions [40]. For a pair of complexes with disjoint protein membership, we draw an undirected edge between them if at least 50% of the possible interactions between one protein from each complex are present in the protein-protein interaction data set. Similarly, for a complex and a single protein, we draw an undirected edge between them if at least 50% of proteins in the complex have a protein-protein interaction with the protein.

#### Relevant interactions curated from literature

The mechanisms for some yeast hits for BMV have been studied in detail [43]–[48]. To leverage this information in our approach, we encode domain knowledge from the literature in the same format as our background network. We have encoded 28 binary interactions among 24 host factors and the external virus node. This set includes the addition of three nodes representing protein complexes, and four interactions between a host component and the virus. Only four of the intracellular interactions were present in the original background network. Table 4 summarizes the interactions derived from literature. Visualizations are available at the supplementary website.

**Table 4:** Interactions from literature.

Cellular process	Source	Nodes	Edges
Ubiquitin-proteasome pathway and lipid production	[43], [48]	8	11
Membrane conformational stability	[47]	4	4
Translation	[44], [46]	12	12
Chaperone proteins	[45]	1	1

Domain knowledge about interactions between yeast and BMV encoded as binary interactions.

### Computational methods

We have developed an integer-linear-programming-based approach to infer a directed subnetwork of interactions that are relevant to virus replication in a host cell. The approach infers subnetworks that have the following properties:

- The subnetwork maximizes the nodes included, subject to constraints.
- A small number of interfaces are predicted; these interfaces are the most downstream nodes in the subnetwork.
- The subnetwork accounts for each hit by providing at least one directed path from the hit to an interface.
- Each relevant edge is assigned a single direction.
- The sign of each relevant edge in the subnetwork is consistent with the phenotypes of its interacting host factors.
- The subnetwork is acyclic.

#### Overview of approach

**Figure 2:**
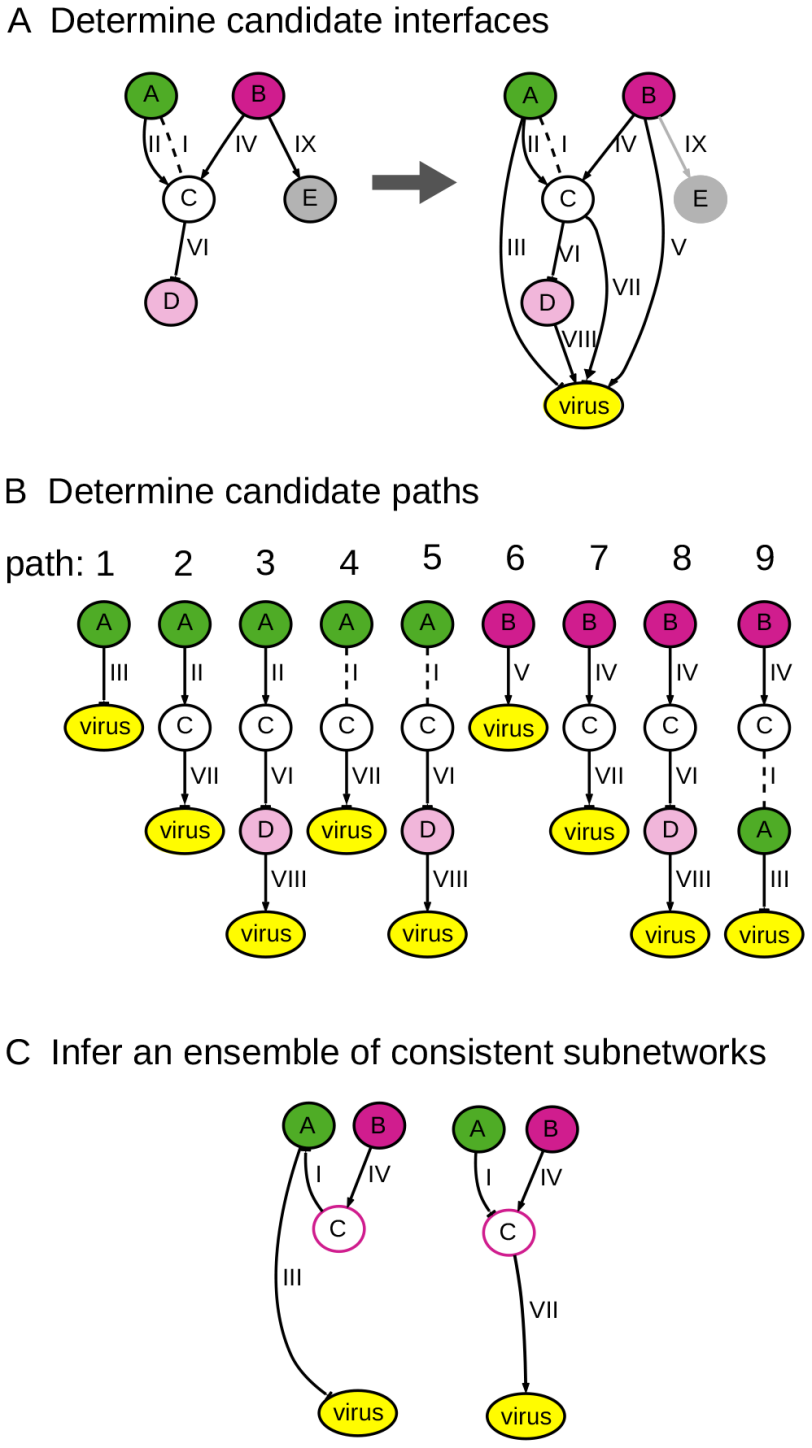
The steps of our subnetwork inference approach. Each edge is shown with a numeric identifier for cross-reference. (**A**) Add a new node to the background network, representing the virus. Add connections between all nodes except **no-effects** to the new virus node, representing the possibility of any host factor having a direct interaction with a viral component. (**B**) For each hit identified by the genome-wide mutant assay, enumerate candidate paths through the background network that could explain it by providing a linear path to the virus node. (**C**) Infer an ensemble of consistent subnetworks. Each subnetwork is a union of paths that accounts for all of the hits and is consistent with virus phenotype data.

In this section we present an overview of the three steps of our approach. Figure 2 illustrates each step applied to a small example background network and set of phenotype labels.

#### Step 1: Determine candidate interfaces

One aspect of the inference procedure is to predict which host factors most directly interact with a viral component. We refer to these as *interfaces*. Before running inference, we identify as *candidate interfaces* all of the host factors that do not have a **no-effect** phenotype label. To represent the possibility of any of these host components interacting with a viral component, we add a special “virus” node to the background network, and add a directed edge from each candidate interface to the virus node. We refer to the edges between host factors and the virus node as *external* edges, and edges between pairs of host factors as *internal* edges. Figure 2(A) depicts the addition of the external edges to the five-node background network shown. No edge is added for node E, which has a **no-effect** phenotype. The set of external edges could be constrained if additional knowledge were available (*e.g*., experimental evidence for specific interactions between host and viral proteins).

#### Step 2: Determine candidate paths

An inferred subnetwork must account for each hit's viral phenotype by either predicting the hit gene to be an interface itself, or by providing a directed, acyclic path to a predicted interface. We enumerate all possible candidate paths of a specified depth leading from each hit to the virus through a candidate interface (as defined in Step 1). Nodes with a **no-effect** phenotype are not included in candidate paths. Nodes with a weak viral phenotype may appear in paths, but are not used as starting points. Figure 2(B) shows the nine candidate paths for the given network.

#### Step 3: Infer an ensemble of consistent, directed subnetworks

An inferred subnetwork comprises a union of directed candidate paths that predicts which host factors are interfaces and provides consistent and directed paths for each hit. We refer to a candidate path that has been chosen to be part of the inferred subnetwork as a *relevant* path. Similarly, we refer to an edge (node) in a relevant path as a *relevant edge (node)*. If an external edge (edge between a host factor and the virus) is predicted to be relevant, the host factor is predicted to be an interface. Inference is a matter of determining the optimal combinations of relevant paths, node phenotypes, interfaces, and edge signs and directions, and is carried out by the IP method.

During inference, the method infers binary viral phenotype labels for all unassayed relevant nodes, and the signs of all relevant edges in cases where they are not specified in the given data. (We do not infer these attributes for nodes and edges that are deemed irrelevant.) While the input data differentiates between weak and strong (hit) viral phenotypes, we predict only the labels **up** or **down** for the unassayed genes that we infer to be relevant. For an edge to be considered relevant, its sign must be consistent with the phenotypes of the interacting nodes. We refer to Figure 2(A) to illustrate this notion of consistency. In the background network, notice that we have evidence that both nodes A and B can activate node C. If edge II is relevant, node C would have the phenotype **up**, to match A's phenotype. However, if edge IV is relevant, node C would have the phenotype **down**, to match B. Since a relevant node can have only one phenotype label, we cannot predict that both edges II and IV are relevant. In addition to using the consistency concept to rule out inconsistently signed edges, we can also use it to infer missing edge signs. If both edges IV and I are relevant, then the inferred phenotype for node C is **down**, and we infer that edge I's sign is inhibition.

The inference process also assigns a direction to all relevant, undirected edges. In the inferred subnetwork, each hit must be able to reach an interface by a directed path. Since a relevant edge can only take one direction, paths 4 and 9 in the example cannot both be predicted to be relevant because they require opposite directions for edge I.

Because of the incompleteness of the background network and experimental data, the space of possible subnetworks that meet all of our requirements is very large. To represent this space, we find an ensemble of subnetworks, where each one corresponds to a different optimal solution to the IP. We initially solve the IP to optimality using a branch-and-cut method [49], and collect multiple solutions by returning to untaken branches. With the ensemble of subnetworks, we thereby assess the confidence in the relevance of a path (node, edge) as the fraction of subnetworks in the ensemble containing that path (node, edge). We measure confidence in the same way for the other inferred quantities: phenotypes, edge signs, and edge directions. Figure 2(C) shows an ensemble of two inferred subnetworks that each account for both hits A and B using one interface.

#### Integer program (IP) variables and notation

Subnetwork inference is performed by solving an integer program (IP), which consists of a set of linear constraints and an objective function, all of which are defined over a set of integer variables that characterize possible subnetworks. The values of some of the variables are determined by the input to the inference process (the phenotypes and background network), whereas others are inferred by the IP. In our implementation, some variables need not be explicitly declared as integer variables because they are constrained such that they can only feasibly take integer values. The implemented program is therefore more precisely a mixed integer linear program.

First, we describe the variables and notation that we use to define the IP. The background network is represented as a graph of nodes 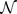, edges 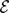, and candidate paths 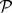. 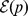 and 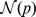 refer to the edges and nodes in a particular candidate path 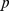, 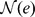 refers to the nodes in a particular edge 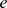, and 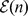 refers to the edges that touch a particular node 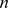. We denote an edge between nodes 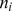 and 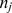 as 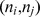.

These sets are further divided into subsets based on experimental data. 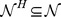 is the set of hit nodes. 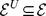 is the set of undirected edges. The complete set of edges 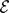 can also be divided into external edges 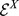, which are added during the execution of our method to provide connections to the virus node, and internal edges 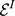, which represent the original background network.

Each node 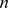 has two variables: 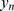, representing whether or not the node is present in any relevant paths, and 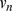, representing its observed or inferred phenotype sign. For hits, we fix 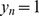 to require that they are present in the inferred subnework. For **down** and **weak-down** genes, we fix 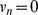; for **up** and **weak-up** genes, we fix 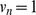. As many as four variables describe each edge. The predicted relevance of an edge 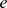 is represented with the variable 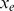, which takes the value 1 if the edge is in at least one relevant path. The sign of an edge is represented by two mutually exclusive variables 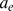 and 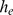. If 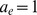 (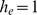), the edge is predicted to be relevant, and inferred to describe an activating (inhibitory) interaction. If an edge is not predicted to be relevant, 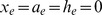. For activating edges given in the background network, 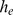 is fixed at 0; similarly, for inhibitory edges, 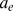 is fixed at 0. Undirected edges also have an associated variable 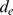, representing the inferred direction of the edge (relative to an arbitrarily chosen canonical direction). If the inferred direction is the same as the canonical direction of the edge, or “forward”, then 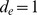; otherwise, 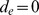. The predicted relevance of a path 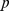 is represented with the variable 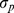, which takes the value 1 if the path is included in the inferred subnetwork, and 0 if it is not.

**Figure 3:**
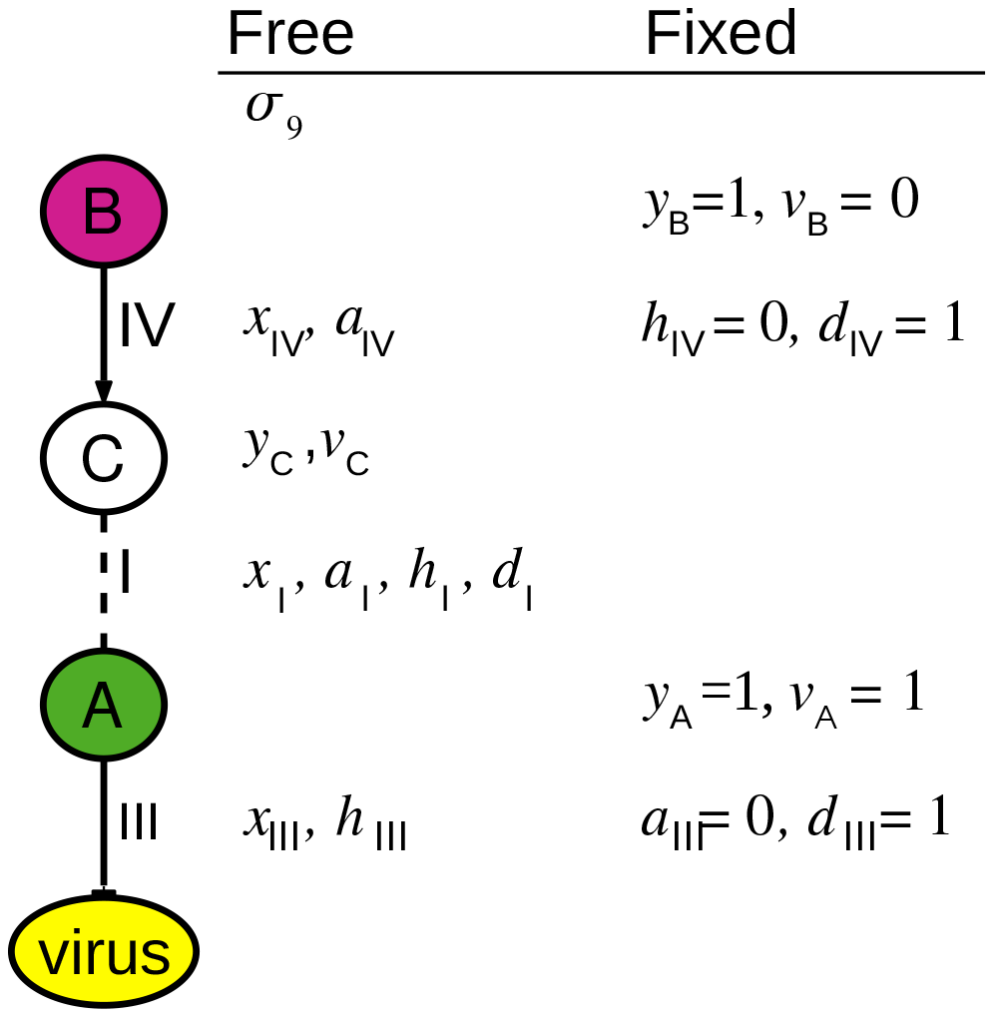
Variables for pathway 9 from Figure 2. The values of some variables are fixed by the data. The values of free variables are determined by the IP.

The variables are summarized in Table 5. Figure 3 shows the variables used to characterize one specific example path.

**Table 5:** Integer program variables.

Network elements	Variable	Interpretation	Values
Paths 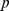	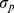	Relevant	no = 0, yes = 1
Edges 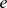	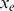	Relevant	no = 0, yes = 1
	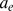	Relevant, activating	no = 0, yes = 1
	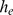	Relevant, inhibiting	no = 0, yes = 1
	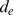	Direction	back = 0, forward = 1
Nodes 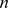	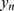	Relevant	no = 0, yes = 1
	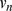	Phenotype	down = 0, up = 1

Binary variables represent the status of nodes, edges, and paths in the network.

#### Diffusion kernel (DK) for node prioritization

To represent the ways in which a hit may modulate the virus through many paths, our inferred subnetworks will generously include consistent nodes and edges. Inspired by the use of graph diffusion kernels to prioritize candidate genes, we use a diffusion kernel method to prioritize non-hit nodes (those with unobserved or weak phenotypes) for inclusion in the subnetwork. (All hits are already required to be included.) The intuition behind this method is that each hit carries some amount of weight that is partially diffused out via its neighbors in the background network. Each node in the network thereby receives a weight according to its proximity and connectivity to the set of hits. This score is used in the objective function of our integer program method.

To calculate the DK scores, we first calculate a regularized Laplacian kernel matrix 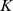 [50], in which the value in each cell represents the proximity and connectivity between two nodes in the graph. The first step is to use the background network to calculate an 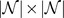 symmetric adjacency matrix 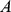. In this matrix, 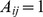 if there is an edge (regardless of direction) between nodes 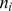 and 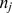 in the background network (internal edges only), and 0 otherwise. Second, we calculate 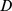, a diagonal degree matrix derived from 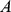, where 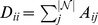. From these, we calculate a normalized Laplacian matrix 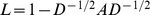. Finally, the kernel matrix is 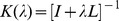.

Next, we use the kernel matrix to calculate how close and connected each node is to the set of hits. We define 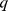 as a binary vector of length 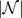 where 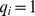 if 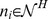 (is a hit) and 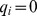 otherwise. Finally, for each node 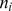, the DK score 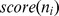 is calculated as 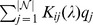.

#### Global objective function and constraints in the IP

The following objective function and two constraints control global properties of the inferred subnetwork.

*Maximize the inclusion of nodes that are proximal and connected to hits*. In order to capture multiple pathways between the hits and the virus, we want to include in the inferred subnetwork the nodes that are most proximal and connected to the hits. Which nodes can be included is limited by the IP's constraints, and so we prioritize nodes using their diffusion kernel score. The objective function of our integer program maximizes the combined score of relevant nodes that are not hits (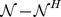).
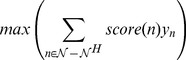



*A small number of interfaces are inferred*. The true number of interfaces is unknown. As a heuristic, we limit the number of interfaces in the inferred subnetwork to a specified integer 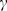. In the inferred subnetwork, we can count the number of interfaces by counting the number of relevant external edges 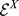, which connect yeast gene nodes to the virus node.
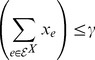



While the objective function tends to maximize the number of nodes in the inferred subnetwork, we can control the size of the subnetwork by restricting the number of interfaces. Depending on the prediction task that the inferred subnetwork will be used for, we may use a more constrained or more generous number of interfaces. If constrained to use only a small number of interfaces, the inference process will identify those interfaces that can explain the most hits. This setting would be appropriate to use when the goal is to predict a high-confidence set of interfaces. On the other hand, allowing more interfaces expands the network and allows for more parallel paths and alternative explanations for hits.

*The proportion of edge signs is constrained*. In the BMV and FHV screens, a hit's phenotype sign (**up** or **down**) is highly correlated with those of its neighbors in the background network. Therefore, we require that the proportion of activating edges in the inferred network is close to a proportion estimated from data. Considering all pairs of hits that interact (under any interaction) in the background network, we record the proportion of pairs with the same phenotype sign. For the BMV data set, this is about 95%; for FHV, it is 100%. The following constraint gives a lower bound 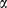 on the proportion of activating internal edges (edges that do not involve the virus node).
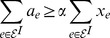
By default, we set this 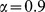 to allow a small deviation from the proportion estimated from the data set. If we did not allow a small number of inhibitory edges into the inferred subnetwork, our inferred subnetworks would not be able to represent connections between two differently-signed hits and the same downstream interface.

#### Local constraints in the IP

Our other subnetwork desiderata are represented as constraints that are used to select which edges and paths are deemed relevant.

*Every hit is included in the inferred subnetwork*. By fixing 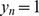 for each hit node 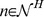, we force the solver to infer a relevant path to account for the hit.
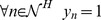



*All edges in a relevant path are relevant*. A relevant edge 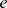 (where 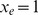) must be in at least one relevant path (for which 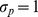); we refer to all paths 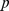 for an edge 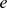 as 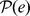. For a relevant path 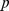, all of its edges 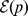 must be relevant.
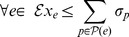


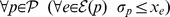



*All nodes in a relevant edge are relevant*. A node 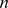 is relevant (that is, 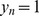) if one if its edges 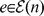 is relevant (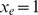). For a relevant edge 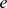, both of its nodes 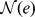 are also relevant.
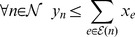


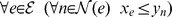



*All relevant edges must be either activating or inhibitory*. The following constraints require that at most one sign variable (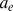 or 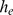) can be equal to 1 for any edge, and that for a relevant edge 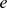 (where 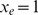), exactly one sign variable must be equal to 1.
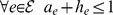


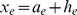



*The sign of a relevant edge is consistent with the phenotypes of the nodes that it connects*. The following set of constraints guide the inference of phenotypes and edge signs for relevant nodes and edges. If a relevant internal edge 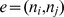 represents activation (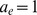), the interacting nodes must have the same phenotype (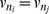).
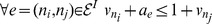


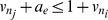



If a relevant internal edge 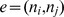 represents inhibition (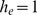), the two interacting nodes must have opposite phenotypes (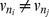).
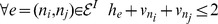


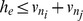



*In a relevant path, all edges are directed toward the interface*. In each relevant path 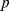, the edges 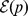 must be oriented toward the virus node at the end of the path. This direction is determined when the candidate path is generated in Step 2, and is given by 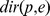. The term including 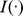, the indicator function, returns 1 if an edge's inferred direction corresponds to the direction required by the path.
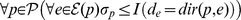



*The inferred subnetwork is acyclic*. While each candidate path is acyclic, it is possible to choose a union of paths that contains cycles. We argue that acyclic inferred subnetworks are more interpretable because they better describe the order of genes in paths and therefore differentiate upstream-acting factors from downstream-acting interfaces. Because searching for and prohibiting all cycles in the inferred subnetwork is an intractable task, we use an approximation that prohibits small cycles. First, we identify sets of edges that induce cycles of a restricted size, and then introduce constraints to the IP so that each possible cycle among relevant nodes must be broken.

We identify cycles among candidate nodes by performing depth-limited, depth-first searches through the candidate nodes in the background network, once per candidate node. If a node is encountered for the second time during the search, then the edges that were taken to get there are saved as a cycle. In our experiments, we search for cycles containing up to three edges. The following constraints require each cycle to be broken. A cycle is broken if either at least one edge in it is inferred to be irrelevant, or if at least one edge is inferred to be directed in the opposite direction of the other edges in the cycle. The precomputed set of possible cycles is 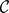, where each cycle 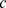 has the set of edges 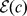. In the second term, the direction of an undirected edge 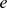 that would complete the cycle 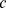 is given by 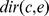.
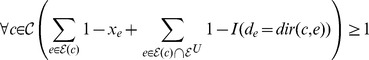



*Interfaces are the most downstream nodes in the subnetwork*. Interfaces are meant to represent the host factors and processes that are closest to a direct interaction with the virus. So, we prohibit a predicted interface 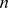 from being inferred to have any other downstream neighbors. Given a particular node 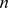, the IP must choose between connecting 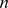 to the virus (thus making it an interface), or inferring any other relevant outgoing edge from 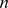. In the following constraints, we refer to the external edge (from 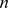 to the virus) using 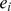, and the internal edges using 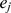. We use separate constraints for directed and undirected internal edges. In the constraint for undirected edges (listed second), the function 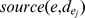 returns the node 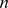 that is the source of the undirected edge 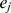 when the edge's direction is set to 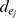.
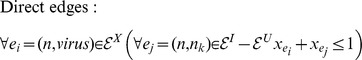


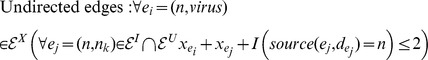



*Specific nodes and edges whose relevance is supported by domain knowledge must be included in the inferred subnetwork*. When we have domain knowledge that a specific host factor or interaction is relevant to viral replication, we can use it to seed the inferred subnetwork by setting its relevance variable to 1 (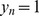 for nodes, 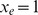 for edges). In the following constraints, 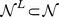 and 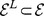 represent the relevant nodes and edges drawn from the literature.
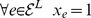


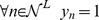



## Results

Although it is not practicable to fully evaluate our inferred subnetworks, we can assess their validity using a number of quantitative and literature-based evaluations.

### Cross-validated phenotype prediction

We first describe an experiment in which we assess the accuracy of our approach in predicting whether test genes with held-aside phenotypes are hits or not. We refer to this as the *hit-prediction* task. Previously, diffusion kernel methods like the one we use in our objective function have been successfully applied to this task, which is also called gene prioritization [31], [33].

Using a leave-one-out methodology, we hold aside the measured phenotype for one gene at a time. The set of genes that are held-aside as test cases for the BMV data set includes 104 hits (49 **up** and 55 **down**) and 1074 **no-effect** genes. The test set for the FHV data set comprises 55 hits (48 **up** and 7 **down**) and 991 **no-effect** genes. We do not test weak-phenotype genes in this evaluation. When a given gene is held aside, it is treated as if its phenotype has the **unobserved** label, meaning that the inference process is used to predict whether or not the gene is relevant, and, if it is predicted to be relevant, its phenotype label. If the test case is included in the set of literature-curated interactions, then all interactions that involve the test case are held aside as well. We also recalculate the diffusion kernel scores for the entire network for each held-aside test case.

To predict the label of a held-aside node, we use our integer programming approach to infer an ensemble of subnetworks. An individual subnetwork may include the held-aside gene and provide a predicted **up** or **down** phenotype for it, or it may exclude the gene. We assess our confidence in whether the gene is a hit or not by determining the fraction of subnetworks in which it is predicted to have an **up** or **down** phenotype. When this fraction is the same for a set of cases, the node scores computed by the kernel are used as a secondary measure of confidence. By varying a threshold on these confidence values, we can plot a precision-recall curve characterizing the predictive accuracy of our method. *Recall* is defined as the fraction of true hits in the test set that are predicted to be hits, and *precision* is defined as the fraction of predicted hits that are truly hits. In this context, we consider precision to be the more important of the two measures, as it is better to to avoid devoting follow-up experiments to false positives.

#### Parameter settings

For all experiments, candidate pathways are limited to a depth of three interactions, and 100 subnetworks are inferred for each ensemble. For the cycle-prohibiting constraint, we compute and disallow cycles of up to three edges. The default setting for 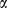, the fraction of inferred activating edges, is 0.9. We initially set 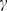, the maximum number of interfaces, to the minimum feasible number that can be used to consistently explain all hits. This is determined for each data set by running a slightly modified version of our IP in which the objective is to minimize the number of interfaces. We perform experiments assessing the effect of raising the level of 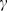 at four additional intervals of 25. For BMV, we perform experiments using 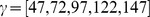; for FHV, we use 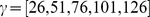. We also assess the effect of the other settings: prohibiting cycles, requiring edges and nodes supported by domain knowledge, and controlling the distribution of edge signs using the parameter 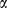. All experiments were performed using GAMS 23.9.3 (for constructing the IP) [51] and the IBM ILOG CPLEX 12.4.0.1 (for solving the IP) [52]. Both are commercial products that are currently available with reduced-cost or free licenses for academic use.

#### Baselines for comparison

We compare our method's precision-recall curve to the curve generated by the diffusion kernel (DK) scores. We also compare against two baselines that use local phenotype information: a hypergeometric test baseline and a nearest neighbor-baseline. In what we call the hypergeometric test baseline, we use the hypergeometric distribution to assign a *p*-value to each held-aside test case gene based on the proportion of hits among its first neighbors relative to the proportion of hits in the entire background network.

To acquire a ranking of the test cases, we sort them in ascending order of their hypergeometric *p*-value. Our second baseline is a naïve nearest-neighbor approach that uses information about both hit and weak viral phenotypes. For each query gene, we count the number of adjacent genes that have either a hit or weak viral phenotype, and rank the test cases in descending order by this count.

We also perform a permutation test in order to estimate our method's ability to predict real viral phenotype hits using randomized input data. The purpose of this test is to estimate how much of our method's predictive accuracy is due to the topological properties (*e.g*., degree, connectivity) of the held-aside genes in the background network, independent of true experimental data. For this test, we infer a subnetwork ensemble for each of 1,000 permuted sets of phenotype labels, and rank actual test cases by their average confidence over the 1,000 inferred subnetwork ensembles. We construct permuted phenotype label sets with approximately the same degree distribution as the original experimental phenotype labels, to control for the effect of degree on the likelihood that a node is predicted to be relevant. To maintain the degree distribution, we draw for each phenotype label a gene from the background network that has the same degree. If fewer than ten genes have the same degree, we expand our consideration to the genes with degree one higher or lower, and continue expanding until we have at least ten to draw from. Among the permuted phenotype label sets for BMV, on average 3.54 true hits (out of 104 in the background network) are retained as permuted hits; for FHV, on average 1.2 true hits (out of 55) are retained.

#### Hit-prediction results

**Figure 4:**
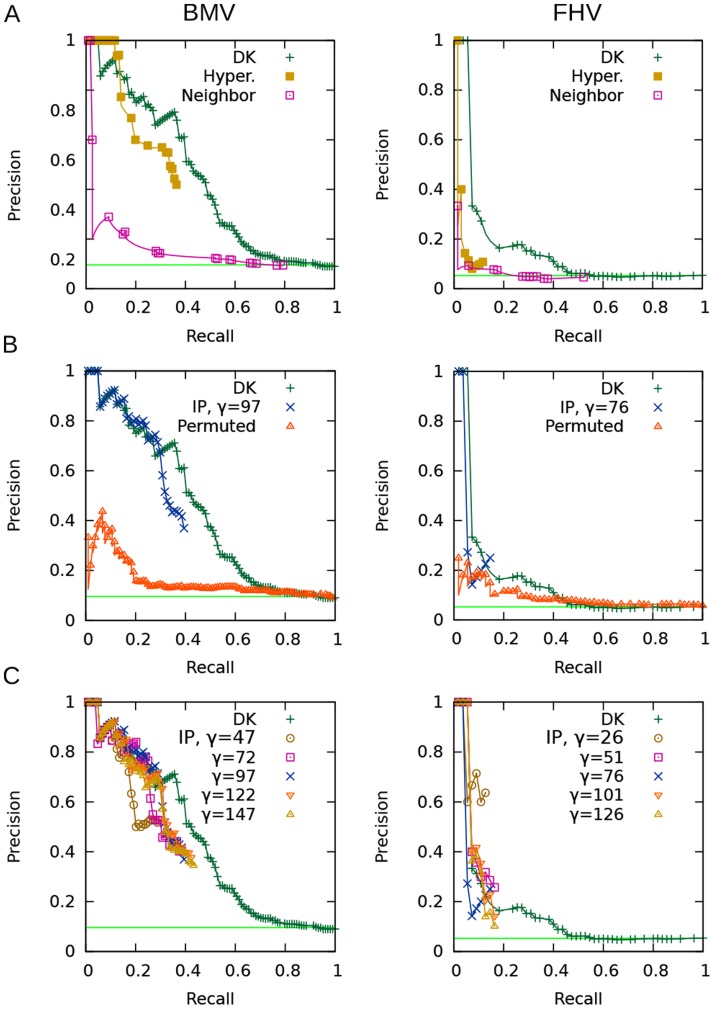
Precision-recall curves for the hit-prediction task. BMV at left, FHV at right. The horizontal line shows precision that would be achieved if all test cases were called hits. (**A**) Comparison of the diffusion kernel method to the naïve baselines. (**B**) Comparison of our IP approach to the diffusion kernel method and to random permutations. (**C**) The effect of varying 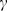, the maximum number of interfaces allowed in the subnetwork inferred by the IP method.

Precision-recall curves for the hit-prediction task are presented in Figure 4. The horizontal line shown in each panel is the fraction of the test set that are hits, thus representing the level of precision that would be achieved by simply predicting that all held-aside genes are hits.

Figure 4(A) compares the diffusion kernel method to the two baselines that employ local phenotype information. For both the BMV and the FHV data sets, the nearest neighbor baseline performs quite poorly: this indicates that, locally, the weak phenotype information is not helpful for making predictions about a node's viral phenotype. The hypergeometric test baseline generally does not perform as well as the diffusion kernel on either data set, although its most highly confident predictions for BMV are more accurate. These results indicate that only a small number of hits can be predicted based only on their local neighborhood, and thus support the use of the diffusion kernel to help identify unassayed genes that might be involved in viral replication. The recall of both of these baselines is bounded by the number of hits that have other hits (or weak-phenotyped genes) among their neighbors.

Figure 4(B) compares our IP method, which uses the diffusion kernel, to the diffusion kernel alone and to the permutation-test baseline. We show the results achieved using the median tested number of interfaces (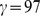 for BMV, 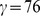 for FHV). (We choose to show the 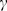 value from the middle of the tested range because, as we discuss later, the method's accuracy does not appear to be very sensitive to the number of allowed interfaces.) In the high-confidence range, our method is able to achieve comparable precision to the kernel method alone, despite the fact that it is making more detailed predictions by specifying interfaces and at least one directed path from each hit to an interface. Both our method and the diffusion kernel method easily surpass the permutation-test baseline's precision. Interestingly, the permutation test's precision is higher than the random guessing line in the low-recall region, suggesting that some hits are more central in the background network compared to **no-effect** genes.

We note that our method does not achieve the same level of recall as the diffusion kernel method. Whereas the diffusion kernel can reach high levels of recall because it propagates nonzero scores to all held-aside genes that are indirectly connected to a hit, the recall of our approach is limited by whether each held-aside gene is included in an inferred subnetwork or not. Our IP can only include a held-aside hit that (i) is used in at least one candidate path for another hit, and (ii) is useful for connecting hits to inferred interfaces. To some extent, we can increase recall by allowing more interfaces in the subnetworks, and by enlarging the number of subnetworks generated in the ensembles. Nevertheless, given the low precision of the diffusion-kernel predictions at high levels of recall, we argue that the recall differences between the two approaches are not of practical significance.

To assess the robustness of our IP with respect to the number of interfaces allowed, we vary 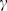 (the maximum number of interfaces) over five values that range from the minimum feasible number to one hundred more. Figure 4(C) presents precision-recall curves for this experiment. For the BMV data set, requiring the minimum number of interfaces results in ensembles that are the least accurate, but the other four values tested produce similar precision to each other, with recall increasing just slightly with 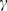. For the FHV data set, the minimum number of interfaces results in higher precision overall in comparison to higher values of 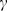, but lower precision in the highest-confidence range. Since the FHV curve represents only a small number of predictions, it is difficult to make strong conclusions based on it. However, the results of the experiment on both data sets suggest that, beyond the minimum allowed, the number of interfaces does not have a large effect on accuracy. For BMV, it appears to be best to use a moderate number of interfaces.

#### Sign-prediction task

As a secondary evaluation, we assess the accuracy of the methods in predicting the correct *sign* of the phenotype (**up, down**) for held-aside hits. We refer to this as the *sign-prediction* task. The methodology for this experiment is largely the same as for the previous one. We hold aside a given hit's phenotype (treating the gene as being **unobserved**), infer an ensemble of 100 subnetworks, and then predict the phenotype sign that is inferred by a plurality of subnetworks. The confidence in a predicted sign is given by the fraction of subnetworks in which the gene is predicted to take that sign. We compare the predictive accuracy of our approach to the diffusion kernel and the baselines considered in the previous experiment. We also tested a variant of the neighbor-voting baseline that employs the notion of consistency described in the Computational Methods section. That is, neighbors connected to the held-aside gene by unsigned and activating edges vote with their own phenotype, but neighbors connected by inhibiting edges vote with the phenotype of opposite sign. The consistency-based baseline performed no better than the simple neighbor-voting methods and thus we report the results only for the original baseline here.

**Figure 5:**
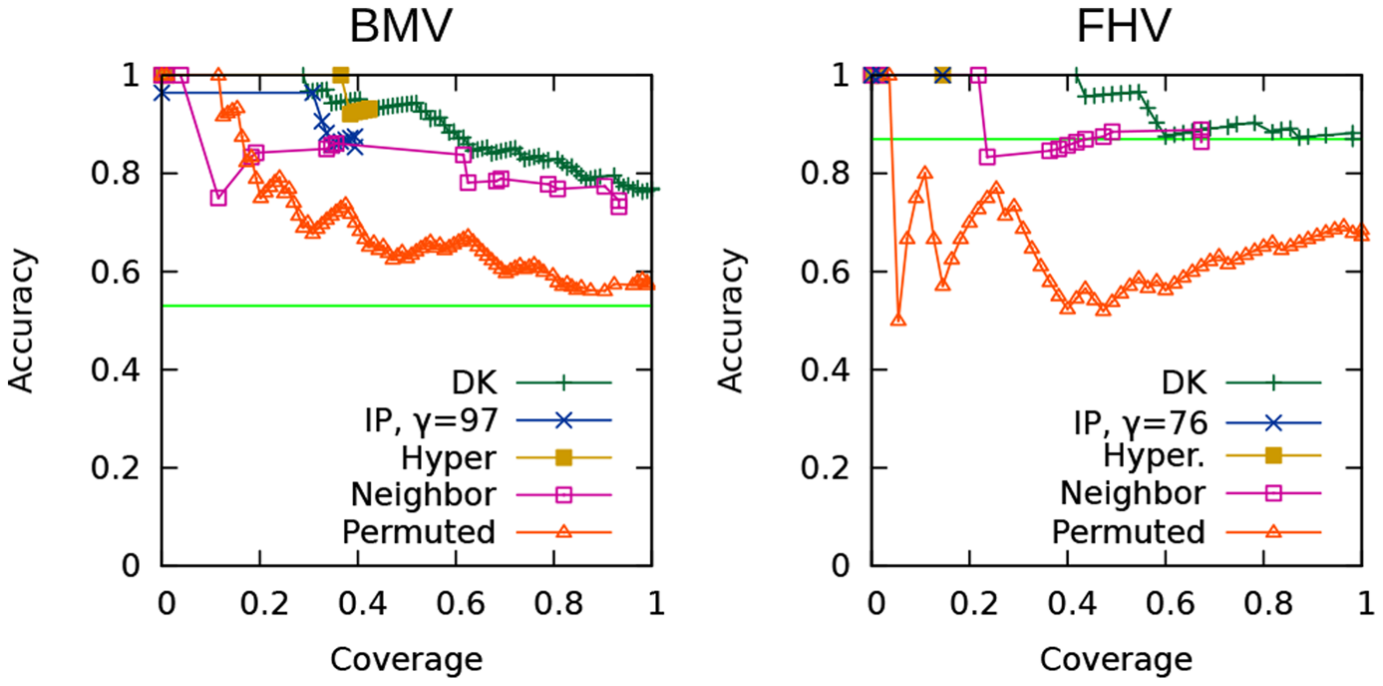
Accuracy-coverage curves for the sign-prediction task. BMV on the left, FHV on the right. The horizontal line indicates the accuracy that would be achieved by assigning the plurality phenotype label to every test case (**down** for BMV, **up** for FHV.)

We construct accuracy-coverage plots for our IP-based approach and both baselines. *Accuracy* is measured as the fraction of phenotype signs correctly predicted, and *coverage* is the fraction of hits (with either **up** and **down** phenotype) for which predictions are made. The hits are sorted by the algorithm's confidence in the predicted phenotype, and accuracy is plotted as coverage increases. The results of this experiment are presented in Figure 5.

For both data sets, the diffusion kernel method is the only one able to make predictions for the entire set of hits, and it achieves high accuracy. Our IP approach matches the diffusion kernel method in the high-confidence range for both data sets. The predictive accuracy of the hypergeometric test is comparable to the IP approach for both data sets. The neighbor-voting baseline is slightly better than our IP method for the FHV data, but inferior for BMV.

#### Stability of the leave-one-out subnetworks

To examine the robustness of our inference method, we compare the ensemble inferred using complete experimental data to the ensembles inferred during the leave-one-out experiments. Specifically, we measure the stability of four types of predictions: (i) which nodes are relevant (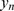), (ii) the phenotype signs of relevant nodes (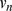 when 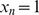), (iii) which nodes are interfaces (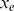 for edges from predicted interfaces to the virus), and (iv) the relevance of nodes that are predicted to be interfaces (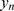, considering only nodes that are ever predicted to be interfaces by any ensemble, but regardless of the confidence in that prediction).

Our method's predictions about node relevance for BMV are highly stable, with average agreement between the complete ensemble and leave-one-out ensembles at or above 90% for ensembles inferred using 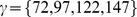 interfaces; for 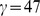 interfaces, the node relevance agreement is slightly lower at 85%. Phenotype sign predictions show slightly lower agreement (80%-84%), as do predictions about which nodes are interfaces (71%–78%). However, predicted interfaces are still likely to be deemed relevant across the set of ensembles, even if they are not as consistently predicted to be interfaces (88%–91%). Overall, predictions for FHV were somewhat less stable than those for BMV, which may be due to the greater connectivity of BMV's hits compared to FHV's. The methodological details and full results for this experiment are available in Text S1 and Table S1-S2.

### Varying components of the IP

As discussed in the Related Work section, several integer programming methods have been developed to infer signalling and regulatory networks from experimental data that comes in the form of source-target pairs. A key aspect of our approach is that it does not assume that targets are given. Instead, it infers the downstream interfaces. Existing IP approaches are therefore not directly applicable to our own task. However, we consider some components of existing methods that can be substituted into our integer program: namely, two alternative objective functions, and one alternative heuristic for inferring edge signs. Additionally, in this section, we explore the effect of varying some of the previously discussed parameters and constraints of our IP.

#### Alternative objective functions

While our objective function maximizes the total diffusion kernel score of relevant nodes, a common goal of other network inference methods is to maximize the number of paths that connect sources and targets. Edges or nodes may also be weighted, giving rise to a weight for each path. Inspired by these methods, particularly by the work by Ourfali *et al*. [12] and Gitter *et al*. [14], we consider two path-based objective functions as alternatives to the node-based objective function that we presented in the Computational Methods section.

**Figure 6:**
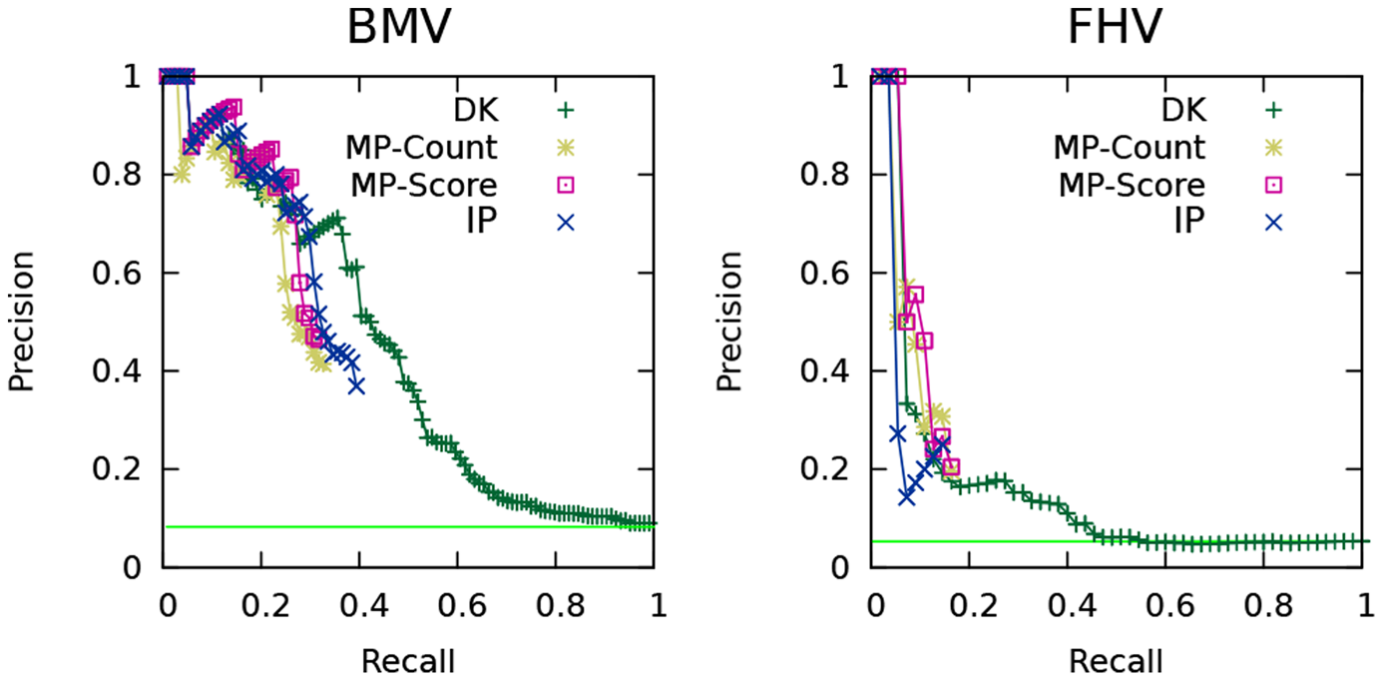
Precision-recall curves for two other objective functions on the hit-prediction task. Comparison of this work's objective function, which maximizes node score (IP), to two alternatives inspired by published methods: maximize path count (MP-Count) and maximize path score (MP-Score). For BMV, the number of interfaces 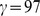. For FHV, 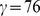.

*Maximize the total count of inferred relevant paths* (MP-Count):
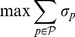



*Maximize the total score of inferred relevant paths* (MP-Score). In advance of inference, we calculate the score of a path as the sum of the diffusion-kernel-derived scores of the nodes in the path:
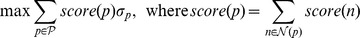



We compare the predictive accuracy of these two path-based objective functions to our node-based objective function using the hit- and sign-prediction tasks that we described previously. The results for the hit-prediction task are shown in Figure 6(A) and (B) for BMV and FHV respectively. The two path-based objective functions perform comparably to our node-based one; thus it does not appear that our IP method is very sensitive to the choice of objective function among the options tested. For the sign-prediction task, again, all three objective functions performed comparably (not shown). Full results for all levels of 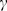 are available in Figure S1 (BMV) and S2 (FHV).

#### Alternative edge sign heuristic

In their SPINE method for inferring signalling networks from source-target pairs, Ourfali *et al* [12] employ the assumption that each node is either a repressor or an activator: that is, all edges leaving from a node must have the same sign. Our own heuristic simply requires that, globally, at least 90% of edges must be activating. To compare the two, we constructed an alternate version of our IP that contains SPINE's heuristic. This is achieved with the introduction of two new variables per node and four new constraints per edge; the details are provided in Text S2.

**Figure 7:**
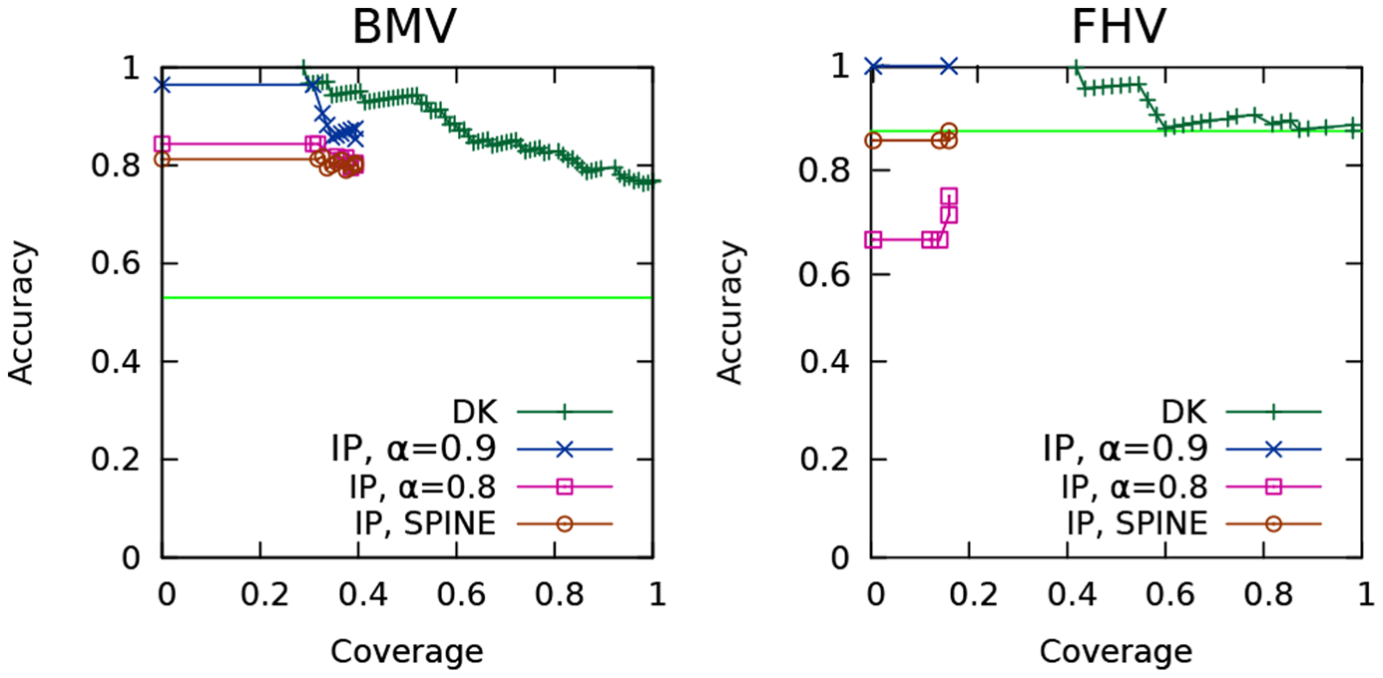
Accuracy-coverage curves for the SPINE heuristic on the sign-prediction task. Comparison of of this work's edge sign heuristic, IP, 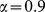, to the heuristic used by the SPINE method[12], IP, SPINE. Also shown is the result for our IP when 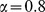.

We use the sign-prediction task to compare our heuristic to SPINE's. As shown in Figure 7, our global constraint results in higher sign-prediction accuracy than SPINE's locally-based constraints. Under the SPINE heuristic, the majority of edges are still inferred to be activating. Among the ensembles that were used to generate the BMV SPINE sign curve in Figure 7, the proportion of activating edges ranges from 0.73–0.84, with a median of 0.79. For BMV, the SPINE heuristic's accuracy appears comparable to that of setting 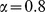. Additional results for all levels of 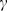 are available in Figure S3 (BMV) and S4 (FHV).

#### Varying parameters and using additional constraints

We also performed additional experiments to measure the effects of other aspects of our method. We summarize the results here, and provide precision-recall curves in Figures S5-S9.

- *Constraining edge signs*. We tested our method under two smaller values of the parameter 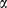, the proportion of activating edges: 0.7 and 0.8. On the sign-prediction task, setting 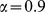 results in the highest accuracy, and accuracy drops as 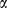 decreases. The hit-prediction accuracy of our method, however, appears to be fairly insensitive to the value of this parameter.
- *Prohibiting cycles in the inferred subnetworks*. We compared precision-recall curves for both data sets and several values of 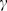, both allowing and disallowing cycles in the inferred network. Disallowing cycles does not appear to have a strong or consistent effect on the method's precision. However, our rationale for prohibiting cycles is based on interpretability considerations.
- *Seeding the subnetwork with edges and interfaces from domain knowledge*. Seeding the subnetwork with literature-curated edges does not appear to have an effect on BMV hit- and sign-prediction accuracy. However, we believe that doing so is qualitatively useful, as it allows our method to make predictions about what additional hits might be explained by already-studied mechanisms.

### Phenotype prediction for unobserved host factors

One motivation for our inference approach is to make predictions about which unassayed host factors may be involved in viral replication. A number of host factors were unable to be assayed using the deletion or doxycycline-repressible mutant libraries, either because the mutant was not part of the library or did not grow under experimental conditions. As these factors cannot be assayed using high-throughput screens, there is a need to identify a high-confidence subset of them for further, lower-throughput experimentation. Toward this end, we use our approach to make predictions about host factors that were not assayed (or not successfully assayed) in the genome-wide BMV screens [1], [4]. We collect an ensemble of 100 inferred subnetworks using all available phenotype data and allowing the use of 97 interfaces. We choose this number of interfaces because it is in the middle of the range tested in our cross-validation experiments, the results of which suggest that prediction accuracy is not significantly affected by a larger number of interfaces. We also seed the network with the literature-curated edges described in the Data section.

Out of 1,821 unassayed host factors in the background network, 221 are predicted to be relevant by any of the 100 inferred subnetworks in the ensemble, and 189 receive 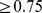 confidence. Of these, 124 represent ORFs (about 9% of unassayed ORFs/putative ORFs), and 65 represent protein complexes (about 14% of represented protein complexes). Here we discuss independent evidence supporting a selection of these predictions.

In numerous cases, the predicted hits include members of pathways of protein complexes known to be involved in BMV replication. In these cases, the inferred subnetworks correctly expanded the relevant complexes with other known components or functional partners that were absent from the given hit sets for technical reasons, such as non-viability of the relevant mutant strain. One example is the inferred inclusion of previously un-implicated components of the cellular ubiquitin-proteasome system, such as the 20S proteasome and components of the 19S regulator complex. While some experimental and literature-curated hits are associated with the proteasome, the predicted hits contribute several more proteasomal proteins. Recent additional experiments, including inhibitor studies and other approaches, have confirmed the involvement of the 20S proteasome, the 19S regulator and other factors in this system in multiple aspects of BMV RNA replication (B.G. and P.A., unpublished results).

**Figure 8:**
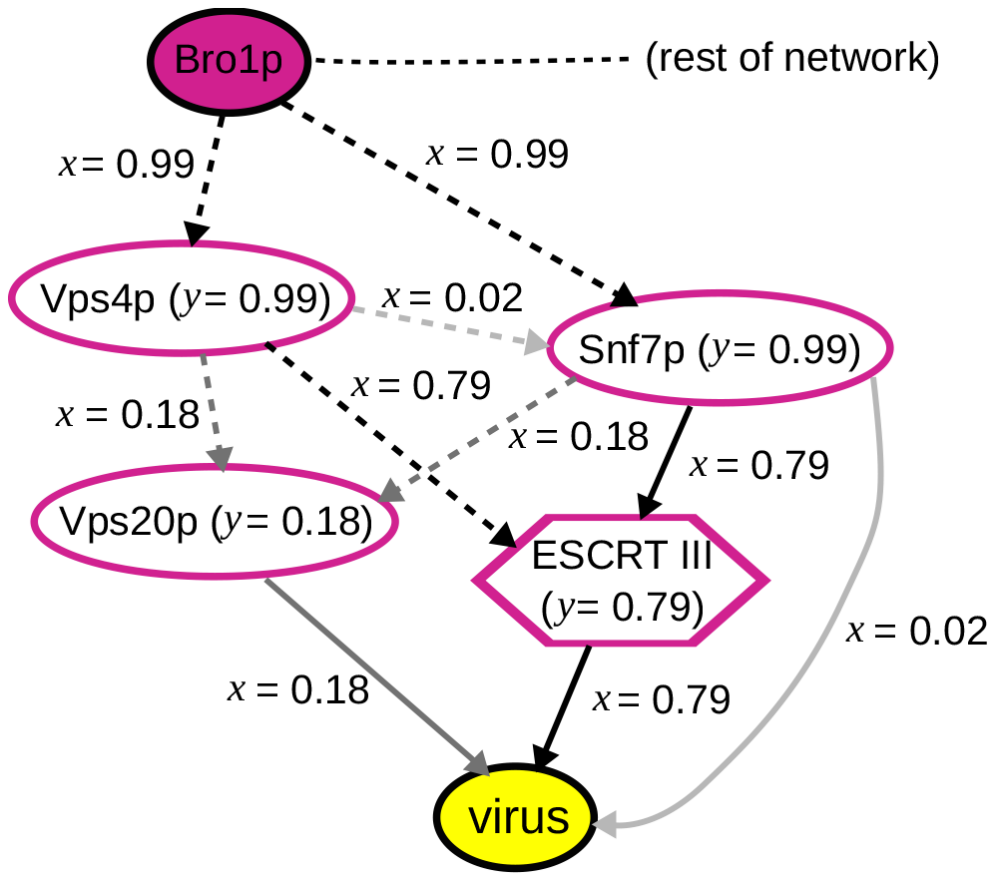
A component from the inferred subnetwork ensemble showing the predicted involvement of Snf7p and Vps4p in viral replication. For predictions made about node and edge relevance, confidence values <1.0 are indicated. For the unassayed nodes, the same phenotype label prediction was made in all solutions in which they appear; similarly, all solutions predicted the same direction for the undirected edges. Dashed edges indicate cases in which the edge's direction was not fixed in the background network. See Figure 1 for a key to the other network elements.

Even more important biological validation of our results emerged from additional experimental studies. For example, our ensemble predicts the involvement of Snf7p and Vps4p, both at 0.99 confidence. These are proteins in the ESCRT pathway, which is involved in membrane bending and scission events in cell division, cell surface receptor down-regulation and other processes [53]. Recent studies initiated independently of the work reported here have confirmed the predicted role of ESCRT pathway, and of Snf7p and Vps4p in particular, in facilitating BMV RNA replication (A. Diaz, X. Wang and P. Ahlquist, manuscript in preparation). The predicted relevant interactions involving Snf7p and Vps4p are shown in Figure 8.

A further example is provided by the inferred involvement of Xrn1p, a protein involved in RNA degradation. An independent study confirmed the strong impact of the gene *XRN1* on BMV replication by showing that a BMV mutant defective in modifying BMV RNA's by the addition of a 5' 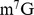 cap could not accumulate RNA in wild type yeast but did so in an *xrn1* 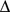 deletion strain [54].

### Host-virus interface predictions

The subnetworks inferred using our method can be used to predict which host factors are closest to a direct interaction with the virus. For this evaluation, we predict a set of high-confidence host interfaces for BMV. The ability of our methods to predict physical interfaces between host protein networks and viral components is constrained by the limits of current background knowledge, as specifically represented by the input background network of interacting host proteins. Because of such external limitations, some predicted interfaces may not represent actual host-virus interfaces, but instead approximate the host component that would most likely connect with an actual interface if the relevant subnetwork were extended to include currently unrecognized interaction partners. We consider support from domain knowledge that the predicted interfaces are plausibly close to a direct interaction with a viral component.

To predict high-confidence interfaces, we infer an ensemble of 100 subnetworks for BMV-yeast interactions, applying the global constraint that only the minimum possible number of interfaces can be used (that is, the smallest number of interfaces such that the IP remains feasible; in this case, 47). We also seed the network with the literature-curated edges described previously, which include four interfaces. Over the entire ensemble, the total number of interfaces used by at least one subnetwork is 51. We designate as “high-confidence” those interfaces that (i) account for more than one hit (other than themselves), (ii) have greater than 0.75 confidence, and, (iii) are predicted to be an interface with an average of at least 0.75 confidence across all of the leave-one-out ensembles inferred using a minimum number of interfaces.

Our method predicts 14 novel high-confidence yeast interfaces for BMV, as shown in Table 6. We assessed these high-confidence interfaces for plausibility based on their annotated function in the Saccharomyces Genome Database [55].

**Table 6:** High-confidence predicted interfaces.

Function or location	Predicted interfaces
Membrane	Nem1p/Spo7p holoenzyme, Set3p complex, Tcb3p, UDP-N-acetylglucosamine complex
Ribosome	Dbp2p
Viral RNA and protein interactions	OCA complex, Ski complex, Smt3p, Ahp1p, 19/22S regulatory complex of proteasome, Cdc34p
mRNA transcription	Gcn5p, Sir4p, Tup1p

Yeast proteins and protein complexes predicted to affect BMV through a direct interaction.

The value of our subnetwork inference method is supported by the observation that several of the predicted 14 high-confidence interfaces are known interactors with BMV components and many more are closely associated with known interactors. Below we discuss available information on several classes of these predicted interfaces.

#### Membrane-associated interfaces

Multiple predicted interface proteins have functions or localization related to endoplasmic reticulum (ER) membranes, the site of BMV genomic RNA replication [56], [57]. Of these, three reside on the ER (the Nem1p/Spo7p holoenzyme, Tcb3p, and the UDP-N-acetylglucosamine transferase complex, which consists of Alg13p and Alg14p). Such proteins may represent anchors for BMV RNA replication complexes, as the mechanisms that localize BMV RNA replication to ER membranes are not completely understood [58]. One of these potential interfaces, Tcb3p, normally resides predominantly on the cortical ER membrane near the cell periphery, rather than on the perinuclear ER membrane that is the major site of BMV RNA replication. However, it was recently shown that BMV RNA replication factor 1a interacts with and induces the relocalization of at least one class of cortical ER membrane proteins, the reticulons, to the perinuclear ER [47]. In addition, the Nem1p/Spo7p phosphatase complex is involved in regulating phospholipid biosynthesis. Regulated synthesis of new lipids is critical to create a specific, expanded membrane compartment essential for RNA replication by BMV [43], [59] and other positive-strand RNA viruses, a number of which were recently shown to interact with lipid synthesis factors to actively promote lipid synthesis [60]. In addition, the Set3p complex is a histone modifier involved in regulating the secretory stress response. This complex might play a role in responding to the extensive occupation of the cell's ER membrane by BMV RNA replication complexes [56], [57].

#### Ribosome-associated interface

One predicted interface is related to ribosomes, which directly interact with BMV genomic and subgenomic RNA to produce all BMV proteins, and thus regulate all steps of BMV replication and gene expression. Dbp2p is involved in processing ribosomal RNA (rRNA) precursors into mature form. The actual yeast-BMV interface might be this rRNA synthesis factor or its rRNA products, which interact with BMV RNAs in their primary role as key ribosomal components. Ribosomal-RNA-related proteins modulate ribosome abundance, which positively and negatively regulates the relative translation levels of different classes of mRNAs, including the competition between polyadenylated cellular mRNAs and non-polyadenylated mRNAs such as those of BMV RNAs [61]. Changes in ribosome synthesis rates, as well as more specific changes, could also alter the specific protein composition of ribosomes, which has can exert dramatic effects on the translation efficiencies of viral mRNAs [62].

#### Interfaces implicated in viral RNA or protein interactions

Additional predicted interface proteins are likely to interact with BMV RNAs or proteins. The Ski complex directs degradation of viral and cellular mRNAs, notably including preferential degradation of non-polyadenylated RNAs like those of BMV [61], [63]. Consistent with direct Ski-mediated degradation of BMV RNAs, knockout of Ski components increases BMV replication [1]. Interestingly, Ski-mediated mRNA degradation involves the exosome, a complex also involved in the rRNA processing discussed above.

The Oca complex (Oca1p, Oca2p, Oca4p-6p and Siw14p) was predicted as an interface because knockouts of each of its genes produced significant BMV replication phenotypes. Two of its subunits, Siw14p and Oca1p, are tyrosine phosphatases. It was suggested previously that these phosphatases may play a role in undermining viral protein phosphorylation events that inhibit RNA replication complex assembly [1], [64].

Finally, multiple predicted interfaces (19/22s regulatory particle of the proteasome, Cdc34p, and Smt3p) are components of the ubiquitin-proteasome system, which covalently modifies proteins to direct their degradation, intracellular trafficking or other purposes. Many viruses encode proteins that interact with this system to modulate viral protein accumulation, targeting or function, or to direct the degradation of interfering cell proteins [65]–[67]. As these precedents include many other positive-strand RNA viruses, BMV may well do the same [68].

#### Interfaces involved in regulation of mRNA transcription

The remaining predicted interfaces are involved in the regulation of mRNA transcription. As BMV is an RNA virus, these proteins are unlikely to be required for the virus' replication in its natural host. Instead, they may be artifacts of the DNA plasmid-directed experimental system used to artificially initiate BMV replication in yeast.

### Impact of provided domain knowledge

One significant advantage of our approach is that it enables domain knowledge to be readily incorporated into the inferred subnetworks. Specifically, the IP can incorporate constraints that represent knowledge about host factors and interactions that are known to be involved in viral replication, thereby influencing decisions about the rest of the subnetwork. These constraints were shown in the Computational Methods section.

Here, we consider the effect of seeding the subnetworks with interactions from specific host pathways that are known to be involved in BMV replication. This set of domain knowledge, which we have elicited from the relevant literature, comprises 28 interactions among 24 host factors. It also specifies several host factors that should be treated as interfaces. For comparison, we also infer BMV subnetwork ensembles that do not use the literature-curated interactions. Seeding the subnetwork with these interactions does not have any apparent effect on hit-prediction accuracy, as we discussed earlier in the Results section. However, the interactions do appear to have an influence on their local neighborhoods. In examining the 97-interface BMV subnetwork ensemble, we observe a small number of cases in which the supplied interactions and interfaces serve to provide “anchors” that allow us to explain other, related hits.

**Figure 9:**
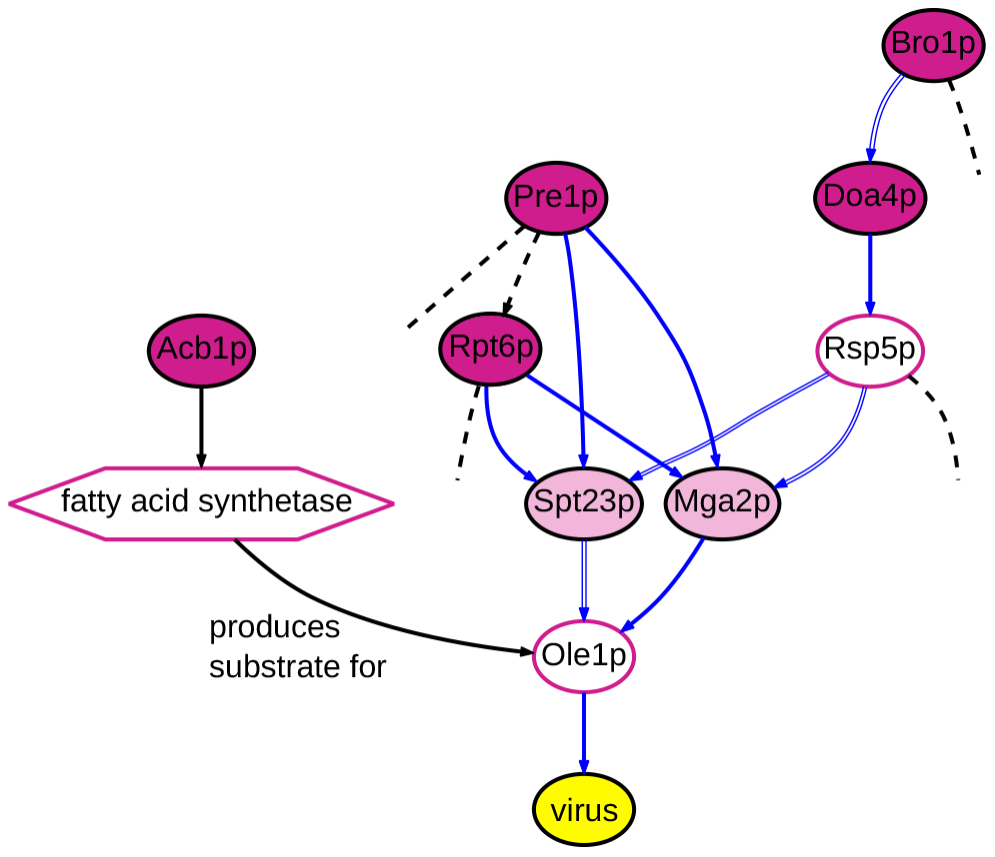
A component from the inferred subnetwork ensemble showing a connection between Acb1 and the literature-extracted ubiquitin-proteasome-system interactions. All node and edge predictions shown have confidence = 1.0 in the ensemble. A dashed edge with no terminal indicates connections to the rest of the subnetwork. Edges extracted from literature are colored blue. Doubled blue edges (as from Rsp5p to Spt23p) indicate literature-extracted edges that were also present in the original background network. See Figure 1 for a key to the other network elements.

One set of edges extracted from the literature connects the ubiquitin-proteasome pathway to membrane synthesis, and specifies that Ole1p is an interface to BMV. The inferred subnetwork identifies a connection between Ole1p, a fatty acid desaturase, and Acb1p, which is involved in transporting newly synthesized fatty acids; the relevant portion of the subnetwork is shown in Figure 9. The connection between the ubiquitin-proteasome pathway and Acb1p was not identified in any subnetwork inferred without the provided literature-based interactions. Furthermore, Ole1p is not inferred to be relevant at all without the provided interactions.

**Figure 10:**
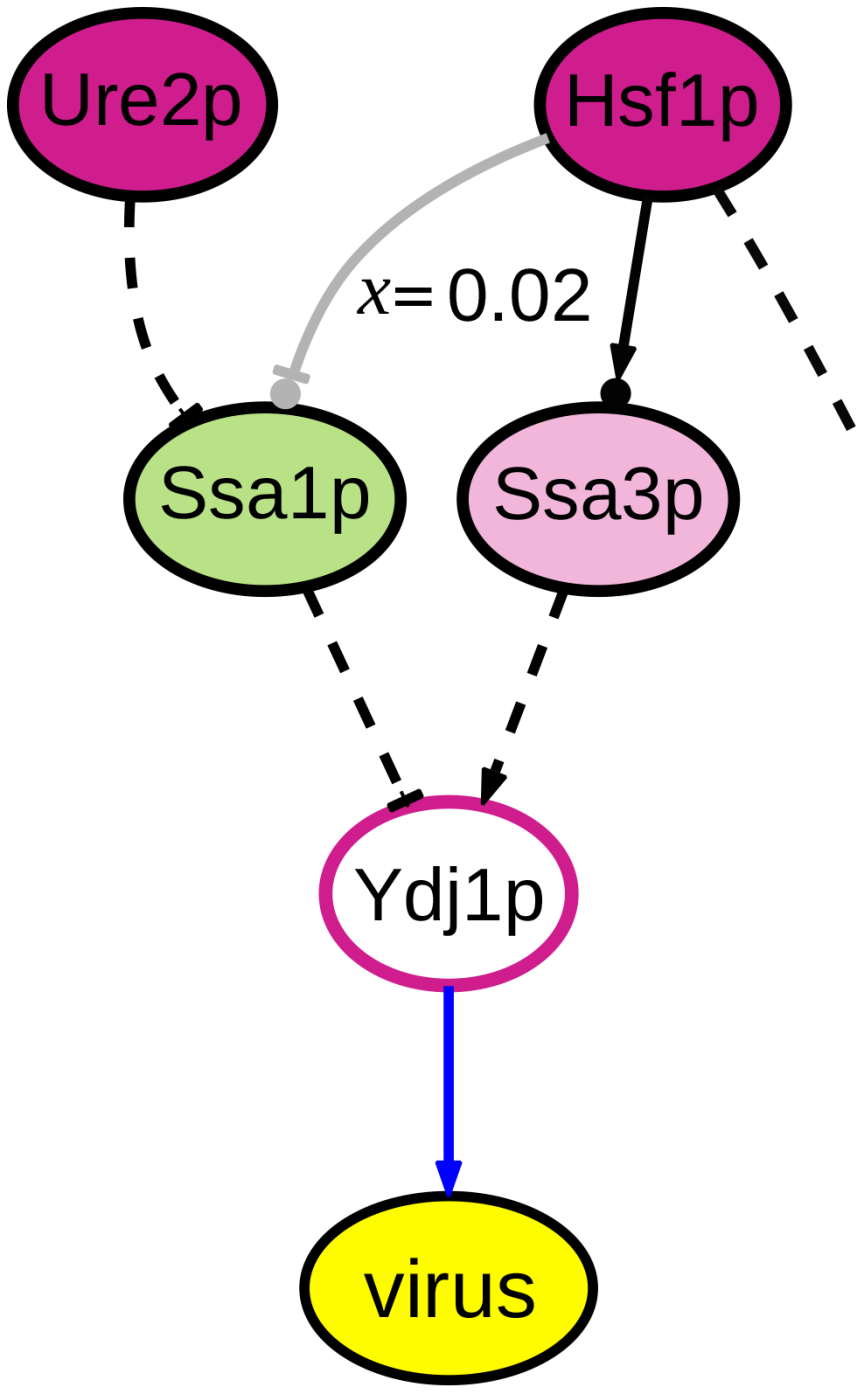
A component from the inferred subnetwork ensemble showing a connection between the literature-identified interface Ydj1p and two hits, Hsf1p and Ure2p. The blue edge from Ydj1p to the virus was originally extracted from literature. See Figure 1 for a key to the other network elements.

Another component from the literature specifies the chaperone protein Ydj1p is an interface. The inferred subnetwork, shown in Figure 10, identifies upstream connections from the hits Hsf1p and Ure2p to Ydj1p, which were not mentioned in the paper discussing Ydj1p's relationship to BMV [45]. These inferred connections demonstrate that the inferred subnetwork can be used to predict relevant connections between well-understood components of the network and host factors that have not yet been studied in detail.

### Gene ontology analysis of inferred BMV subnetwork

To supplement our manual analysis of predicted hits and interfaces, we employ the Model Based Gene-Set Analysis (MGSA) tool [69] to evaluate the ability of the inferred subnetwork to better identify relevant functional categories than an analysis of the experimental hits alone. The MGSA method uses a Bayesian network to analyze the representation of all GO terms in a gene set at once. As output, it provides the marginal probability that each GO term accounts for the input gene set. We use MGSA to analyze first the experimental hits and literature-derived relevant genes for BMV that are present in the background network, and second, the experimental hits combined with the predicted hits from the 97-interface inferred subnetwork. We use a probability threshold of 0.25 because we are willing to tolerate a degree of redundancy in the results, in exchange for the identification of a thorough list of representative GO terms.

We further assess the significance of each returned GO term by comparison to the subnetworks inferred from random data. For each GO term, we generate a *p*-value as the proportion of random subnetworks for which MGSA gives a greater or equal probability.

Table 7 presents the GO terms returned by MGSA with probability 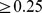 for the combined set of experimental and predicted hits. The “Experimental Hits” columns show the number of experimental hits associated with each GO term, and MGSA's probability that the GO term explains the experimental hits alone. Similarly, the “Predicted Hits” columns show the number of additional predicted hits associated with the GO term, and MGSA's probability that the GO term explains the combined experimental and predicted hit set. The “*p*-value” column shows the proportion of random subnetworks with equal or greater probability for the GO term as compared to the inferred subnetwork, with asterisks indicating 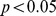.

**Table 7:** Gene Ontology terms represented by both experimental and predicted BMV hits.

			Experimental hits		Predicted hits			
GO ID	Description	Size	Count	Prob.	Count	Prob.	*p*-value	
32447	protein urmylation	7	6	0.975	0	0.860	0.002	*
33588	Elongator holoenzyme complex	6	5	0.974	1	0.783	0.084	
55087	Ski complex	4	4	0.961	0	0.783	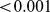	*
32874	positive regulation of stress-activated MAPK cascade	3	3	0.954	0	0.694	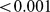	*
71782	endoplasmic reticulum tubular network	3	3	0.848	0	0.685	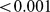	*
5688	U6 snRNP	8	5	0.704	0	0.593	0.216	
445	THO complex part of transcription export complex	4	2	0.265	1	0.376	0.031	*
446	nucleoplasmic THO complex	4	2	0.307	1	0.363	0.038	*
32784	regulation of DNA-dependent transcription, elongation	3	2	0.288	1	0.292	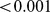	*
5732	small nucleolar ribonucleoprotein complex	9	5	0.260	0	0.272	0.049	*
3724	RNA helicase activity	5	3	0.356	1	0.264	0.005	*
71072	negative regulation of phospholipid biosynthetic process	2	2	0.369	0	0.262	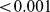	*
36083	positive regulation of unsaturated fatty acid biosynthetic process by positive regulation of transcription from RNA Pol II promoter	2	2	0.321	0	0.251	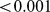	*

GO terms that MGSA returns for both BMV experimental hits alone and for the inferred BMV subnetwork (with probability 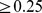). In the “Experimental hits” column are shown the number of hits associated with the GO term, and MGSA's probability that the GO term explains the experimental hit set alone. In the “Predicted hits” column are shown the number of predicted hits associated with the GO term, and MGSA's probability that the GO term explains the combined experimental and predicted hit set. The column “*p*-value” shows the proportion of random subnetworks for which the MGSA probability of the GO term is greater than or equal to that of the inferred subnetwork; asterisks indicate 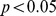.

As shown in Table 8, an additional 15 GO terms are identified by MGSA for the combined hit set, but are not identified for the experimental hits alone. A number of these GO terms represent only predicted hits. Eight of the GO terms receive a 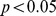 from the random subnetwork analysis. This result indicates that our subnetwork inference method predicts hits that (i) are useful for amplifying weak functional signals among the experimental hits, and (ii) are among themselves functionally coherent. Several of the amplified GO terms represent protein complexes or pathways that are recognized for their role in BMV replication. Deadenylation-dependent mRNA decapping factors are also known to be relevant [44], and the perinuclear region of the cytoplasm is the cellular location in which BMV replicates [56]. Among the novel GO terms that contain no experimental hits are represented specific parts of the ubiquitin-proteasome system and ribosome synthesis, both of which we have noted are relevant to BMV replication.

**Table 8:** Additional Gene Ontology terms represented by the inferred BMV subnetwork.

			Experimental hits		Predicted hits			
GO ID	Description	Size	Count	Prob.	Count	Prob.	*p*-value	
42790	transcription of nuclear large rRNA transcript from RNA polymerase I promoter	17	3	0.022	5	0.977	0.001	*
502	proteasome complex	43	7	0.057	24	0.947	0.083	
70847	core mediator complex	20	0	–	8	0.873	0.301	
290	deadenylation-dependent decapping of nuclear-transcribed mRNA	9	3	0.143	2	0.798	0.021	*
48471	perinuclear region of cytoplasm	12	3	0.036	2	0.636	0.003	*
124	SAGA complex	20	0	–	7	0.631	0.105	
71629	cytoplasm-associated proteasomal ubiquitin-dependent protein catabolic process	4	0	–	3	0.621	0.001	*
34455	t-UTP complex	7	0	–	4	0.470	0.104	
30015	CCR4-NOT core complex	9	0	–	4	0.450	0.213	
33553	rDNA heterochromatin	9	0	–	5	0.430	0.014	*
34388	Pwp2p-containing subcomplex of 90S preribosome	6	0	–	3	0.426	0.100	
31146	SCF-dependent proteasomal ubiquitin-dependent protein catabolic process	17	0	–	6	0.390	0.104	
6750	glutathione biosynthetic process	2	0	–	2	0.315	0.016	*
6283	transcription-coupled nucleotide- excision repair	9	0	–	4	0.291	0.017	*
7584	response to nutrient	2	0	–	2	0.288	0.002	*

GO terms that MGSA returns for the inferred BMV subnetwork, but not for the BMV experimental hits alone (with probability 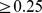). In the “Experimental hits” column are shown the number of hits associated with the GO term, and MGSA's probability that the GO term explains the experimental hit set alone. In the “Predicted hits” column are shown the number of predicted hits associated with the GO term, and MGSA's probability that the GO term explains the combined experimental and predicted hit set. The column “*p*-value” shows the proportion of random subnetworks for which the MGSA probability of the GO term is greater than or equal to that of the inferred subnetwork; asterisks indicate 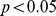.

### Permutation analysis of inferred relevant complexes

One advantage of our method is that we explicitly include protein complexes as nodes in our background network. We propose that doing so allows the inferred subnetworks to provide useful information about cooperative interactions between proteins. We use two Monte Carlo tests to assess the degree to which the representation of complexes among inferred subnetworks, and specifically among inferred interfaces, is due to (i) topological properties of the background network and inference procedure, independent of the experimental data, and (ii) properties of the experimental data, independent of the inference procedure. The details of these experiments are available in Text S3, with results in Tables S3-S4.

Considering predicted relevant complexes in the 97-interface BMV subnetwork, 17 out of 65 predicted relevant complexes receive a *p*-value below 0.05 from either Monte Carlo test. Of the high-confidence, predicted BMV-yeast interfaces that are protein complexes or are members of complexes, five out of the eight receive a *p*-value below 0.05 from either test. These results indicate that the representation of many complexes by our inferred subnetworks are well-supported by predicted hits and are not likely to be artifacts of the background network or chance.

## Discussion

We have presented an approach that aims to elucidate how viruses exploit their host cells. Our approach uses known host intracellular interactions to infer ensembles of directed subnetworks which provide consistent explanations for phenotypes measured in genome-wide loss-of-function assays. This approach is able to represent a rich set of interaction types, in addition to domain knowledge about specific interactions that are known to be relevant. By inferring an ensemble of subnetworks, the approach is able to quantify its certainty about the relevance of various genes and interactions.

The value of the subnetworks inferred by our method is that they can be used to (i) predict which unassayed genes may be involved in viral replication, (ii) interpret the role of each hit in modulating the virus, and (iii) guide further experimentation. Our empirical evaluation demonstrates that, using a gene-prioritization method as a sub-component, our method is able to predict phenotypes for unassayed genes with accuracy that is comparable to the gene-prioritization method alone. We also used our method to predict host-virus interfaces and additional relevant host genes for Brome Mosaic Virus, and performed a literature-based analysis of the predicted relevant host factors. While additional experimentation is necessary to confirm our predictions, a number of them are supported by domain knowledge. Among the predicted interfaces, many are known to bind or modify RNA, localize to the site of viral replication, or act in processes that have been previously identified as involved in viral replication. Similarly, many predicted hits are members of known relevant complexes, and a few are supported by independent experiments. These results are also supported by a Gene Ontology analysis which showed that our inferred subnetworks identify more relevant functional categories than the experimental data alone. Our experiments also demonstrated that the predictions made by our inferred networks have high levels of stability given small changes to the input data.

There are a number of promising directions in which we plan to extend this work. Among them are applying the method to RNAi studies in more complex host networks and incorporating literature-extracted interactions into the background network.

Our supplementary website is located at http://www.biostat.wisc.edu/~craven/chasman_host_virus/. There we provide integer program code and data in the GAMS language, and visualizations of the background network and inferred BMV subnetworks as Cytoscape [70] files.

## Supporting Information

Figure S1
**Precision-recall and accuracy-coverage curves for path-based objective functions; BMV dataset.** Results are provided at all levels of 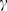 (the number of interfaces).(PDF)

Figure S2
**Precision-recall and accuracy-coverage curves for path-based objective functions; FHV dataset.** Results are provided at all levels of 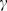 (the number of interfaces).(PDF)

Figure S3
**Precision-recall and accuracy-coverage curves for the SPINE phenotype-sign heuristic; BMV dataset.** Results are provided at all levels of 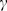 (the number of interfaces).(PDF)

Figure S4
**Precision-recall and accuracy-coverage curves for the SPINE phenotype-sign heuristic; FHV dataset.** Results are provided at all levels of 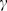 (the number of interfaces).(PDF)

Figure S5
**Precision-recall and accuracy-coverage curves showing the effect of varying** 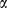 **; BMV dataset.** Results are provided for at all levels of 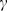 (the number of interfaces).(PDF)

Figure S6
**Precision-recall and accuracy-coverage curves showing the effect of varying** 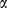 **; FHV dataset.** Results are provided for at all levels of 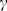 (the number of interfaces).(PDF)

Figure S7
**Precision-recall and accuracy-coverage curves assessing accuracy of the cycle-prohibiting constraint; BMV dataset.** Results are provided at all levels of 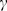 (the number of interfaces).(PDF)

Figure S8
**Precision-recall and accuracy-coverage curves assessing the accuracy of the cycle-prohibiting constraint; FHV dataset.** Results are provided all levels of 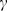 (the number of interfaces).(PDF)

Figure S9
**Precision-recall and accuracy-coverage curves assessing the accuracy of literature-curated interactions.** Results are provided for BMV at all levels of 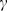 (the number of interfaces).(PDF)

Table S1
**Stability of leave-one-out inferred subnetworks.** Stability of predictions for all settings of 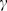.(PDF)

Table S2 **Sizes of inferred subnetworks.** Average counts of weak and unassayed host factors that are predicted to be relevant by the leave-one-out ensembles.
(PDF)

Table S3 **Enriched, predicted relevant, protein complexes.** List of protein complexes predicted to be relevant and supported by permutation analyses.
(PDF)

Table S4 **Interfaces accounted for by enriched complexes.** List of high-confidence interfaces that are or are represented by significantly-enriched protein complexes.
(PDF)

Text S1 **Stability analysis of inferred subnetworks.** Analysis of the stability of the predictions made by the cross-validation ensembles.
(PDF)

Text S2 **SPINE edge-sign prediction heuristic.** Description of the IP variables and constraints used to implement the SPINE-based edge sign prediction heuristic.
(PDF)

Text S3 **Permutation analysis of inferred relevant complexes.** Further analysis of predicted relevant protein complexes.
(PDF)
