# Supplementary material for: Inferring Host Gene Subnetworks Involved in Viral Replication
Source: PLoS Comput Biol. 2014 May 29;10(5):e1003626. doi: 10.1371/journal.pcbi.1003626 (PMC4038467; doi:10.1371/journal.pcbi.1003626)

BMV Hit Prediction:
Comparison between objective functions (our work: IP)
(cycles disallowed, α=0.9, using literature interactions)

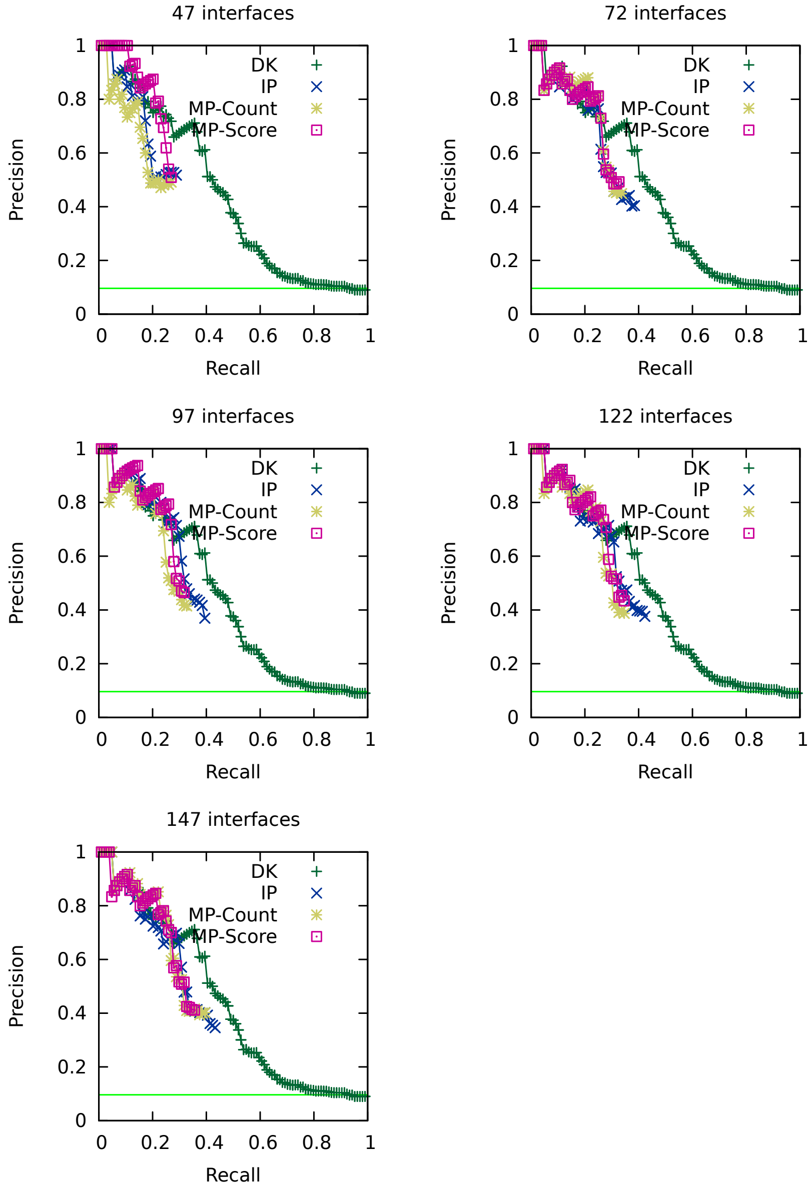

BMV Sign Prediction:
Comparison between objective functions (our work: IP)
(cycles disallowed, α=0.9, using literature interactions)

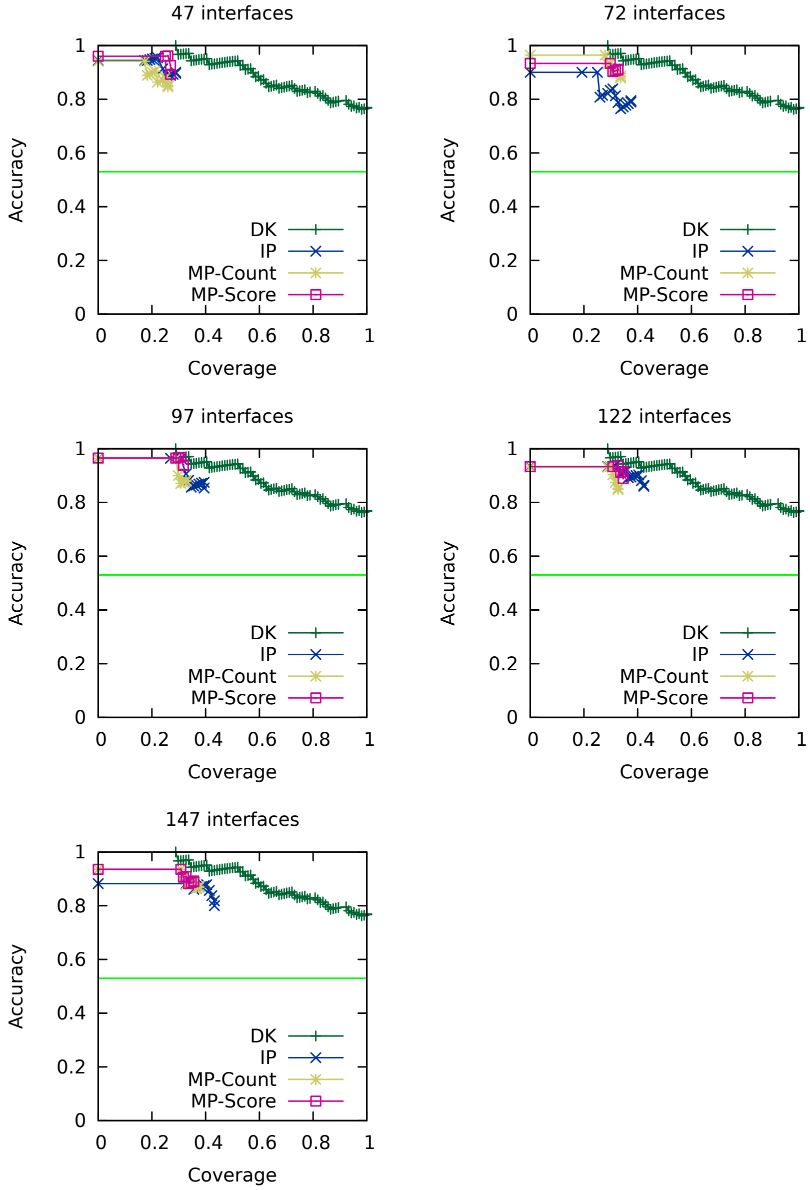

Supplement: Figure S1 — Precision-recall and accuracy-coverage curves for path-based objective functions; BMV dataset. Results are provided at all levels of (the number of interfaces). (PDF) [file pcbi.1003626.s001.pdf]
