# Supplementary material for: Inferring Host Gene Subnetworks Involved in Viral Replication
Source: PLoS Comput Biol. 2014 May 29;10(5):e1003626. doi: 10.1371/journal.pcbi.1003626 (PMC4038467; doi:10.1371/journal.pcbi.1003626)
Supplement: Figure S2 — Precision-recall and accuracy-coverage curves for path-based objective functions; FHV dataset. Results are provided at all levels of (the number of interfaces). (PDF) [file pcbi.1003626.s002.pdf]

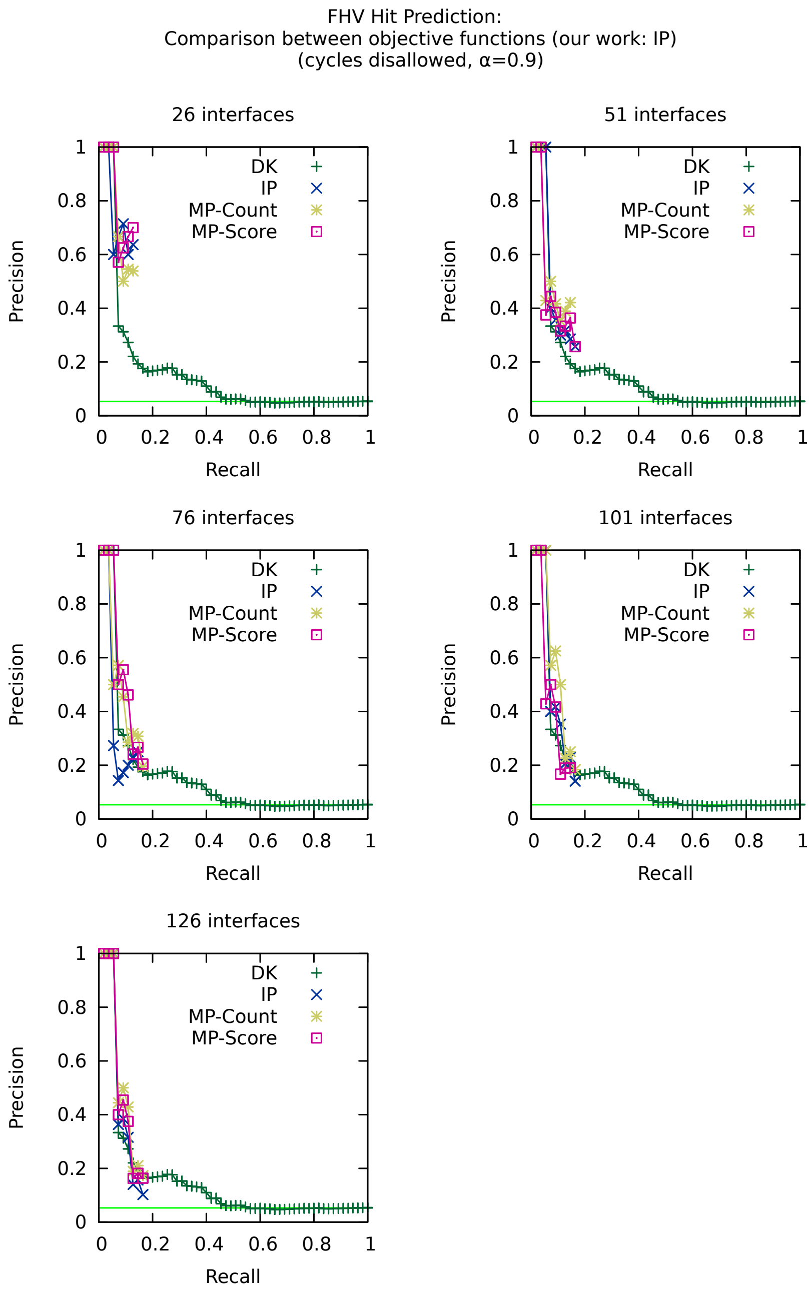
FHV Hit Prediction:
Comparison between objective functions (our work: IP)
(cycles disallowed, α=0.9)
26 interfaces
51 interfaces
76 interfaces
101 interfaces
126 interfaces
Precision
Recall
DK
IP
MP-Count
MP-Score
0
0.2
0.4
0.6
0.8
1

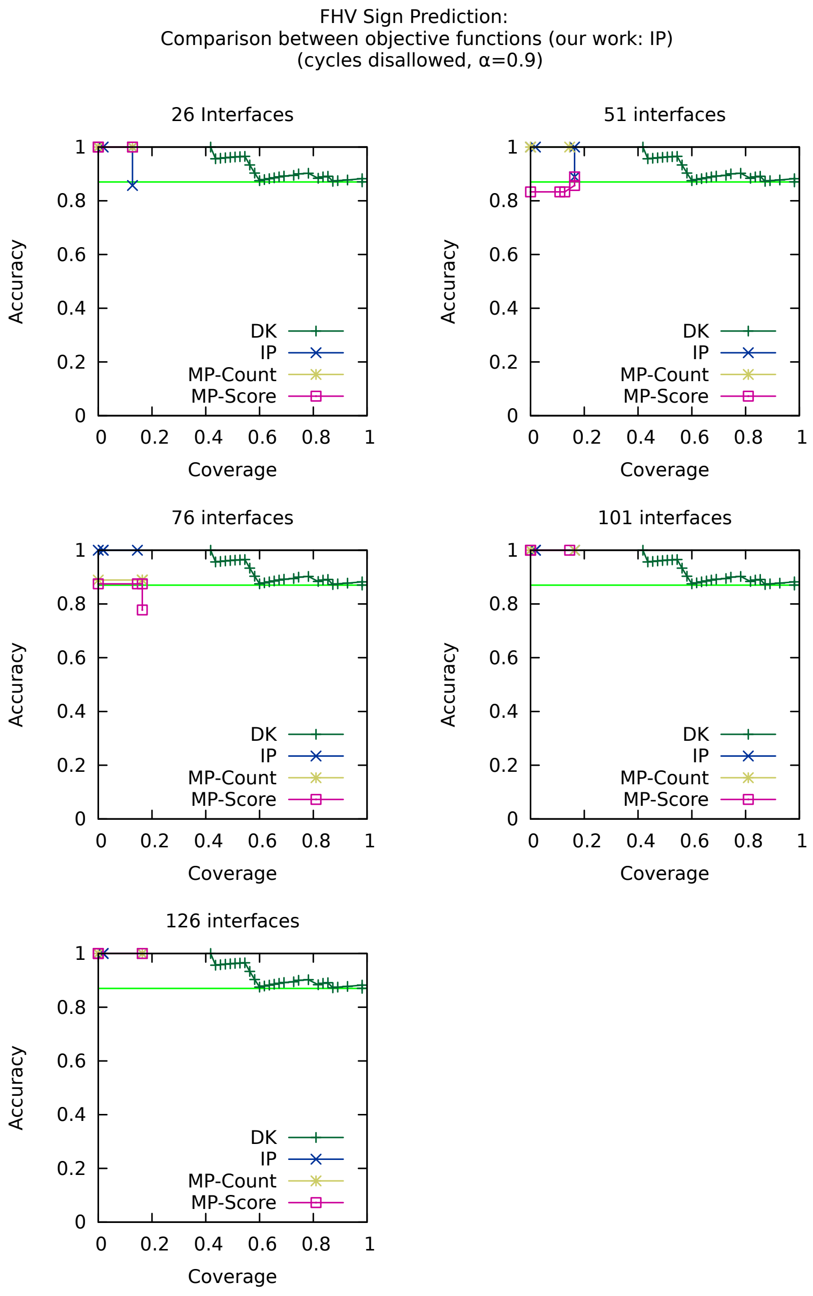
FHV Sign Prediction:
Comparison between objective functions (our work: IP)
(cycles disallowed, α=0.9)
26 Interfaces
51 interfaces
76 interfaces
101 interfaces
126 interfaces
Accuracy
Coverage
DK
IP
MP-Count
MP-Score
0
0.2
0.4
0.6
0.8
1
