# Supplementary material for: Inferring Host Gene Subnetworks Involved in Viral Replication
Source: PLoS Comput Biol. 2014 May 29;10(5):e1003626. doi: 10.1371/journal.pcbi.1003626 (PMC4038467; doi:10.1371/journal.pcbi.1003626)
Supplement: Figure S4 — Precision-recall and accuracy-coverage curves for the SPINE phenotype-sign heuristic; FHV dataset. Results are provided at all levels of (the number of interfaces). (PDF) [file pcbi.1003626.s004.pdf]

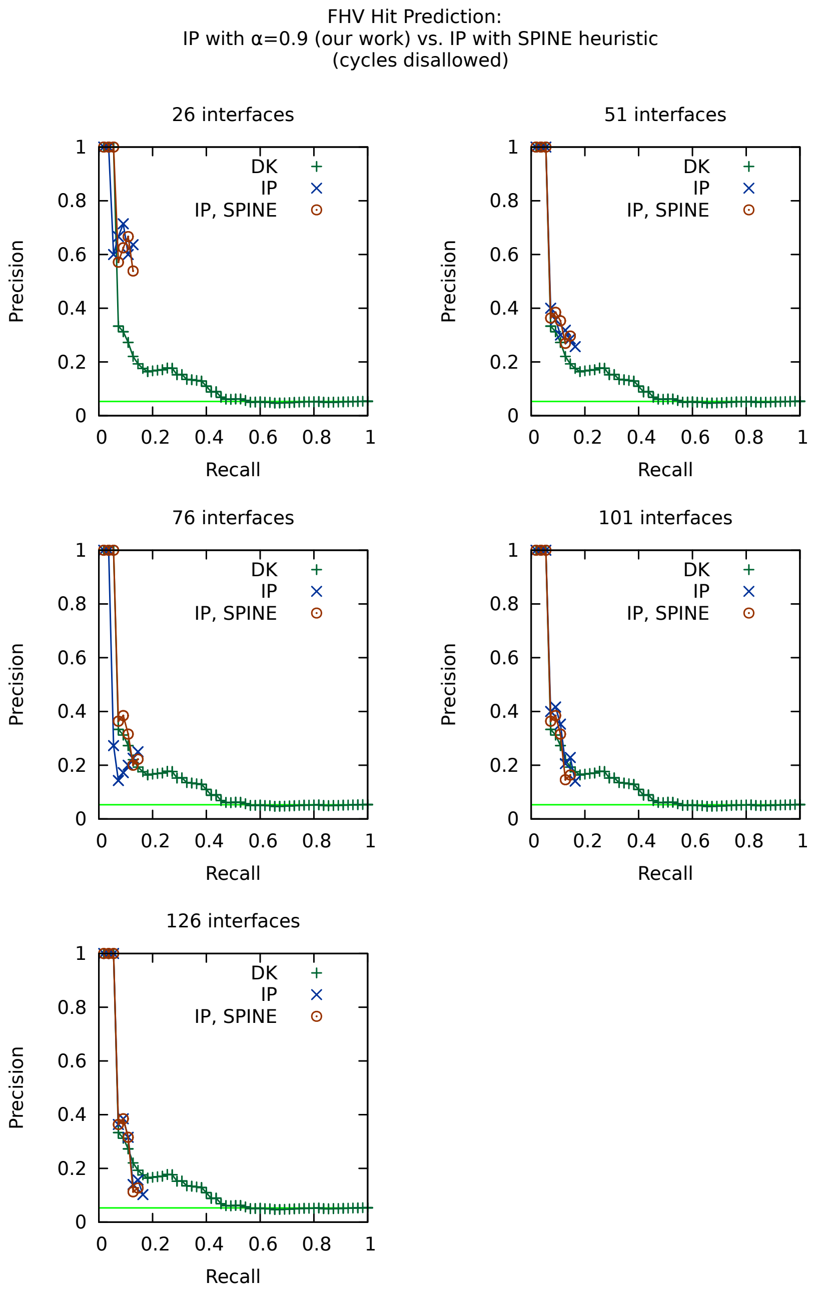

FHV Hit Prediction:
IP with α=0.9 (our work) vs. IP with SPINE heuristic
(cycles disallowed)
26 interfaces
51 interfaces
76 interfaces
101 interfaces
126 interfaces
DK
IP
IP, SPINE
Precision
Recall

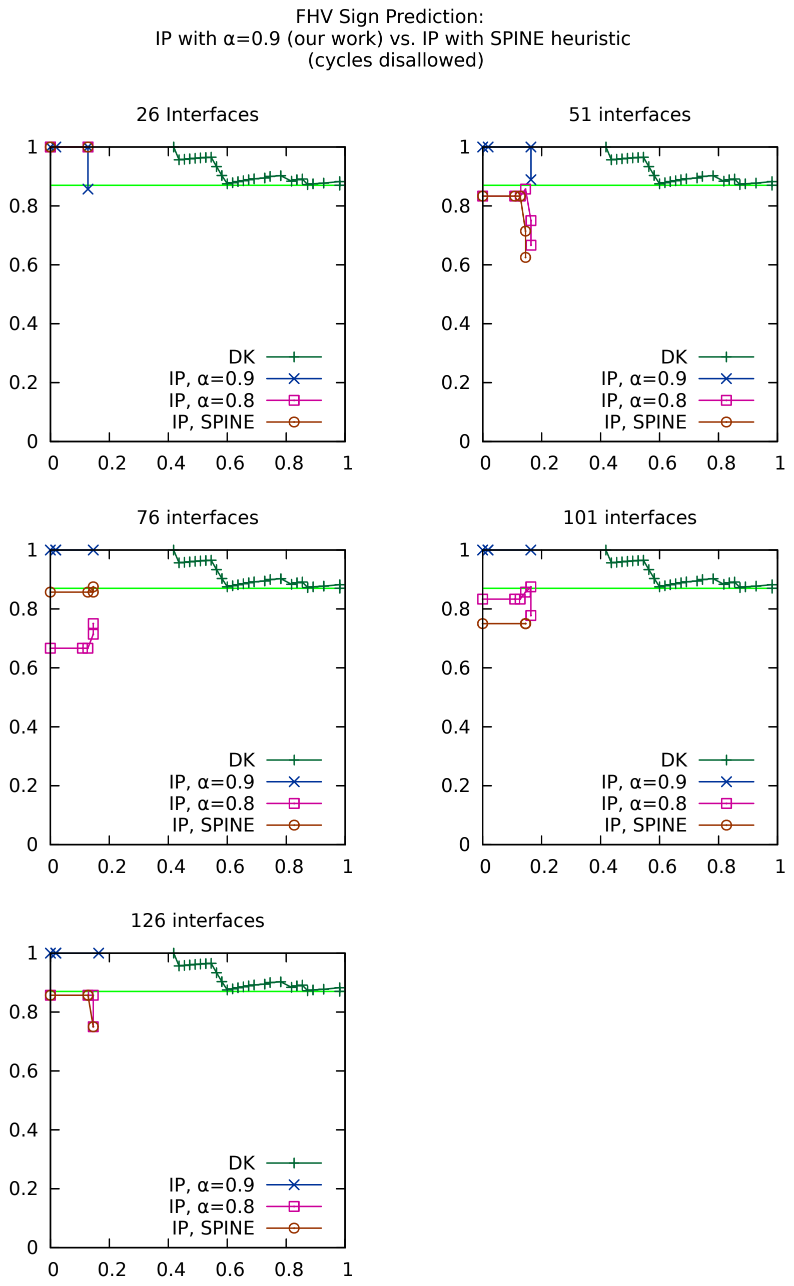

FHV Sign Prediction:
IP with α=0.9 (our work) vs. IP with SPINE heuristic
(cycles disallowed)
26 Interfaces
51 interfaces
76 interfaces
101 interfaces
126 interfaces
DK
IP, α=0.9
IP, α=0.8
IP, SPINE
