# Supplementary material for: Inferring Host Gene Subnetworks Involved in Viral Replication
Source: PLoS Comput Biol. 2014 May 29;10(5):e1003626. doi: 10.1371/journal.pcbi.1003626 (PMC4038467; doi:10.1371/journal.pcbi.1003626)

BMV Hit Prediction:
Varying α
(using literature interactions, cycles disallowed)

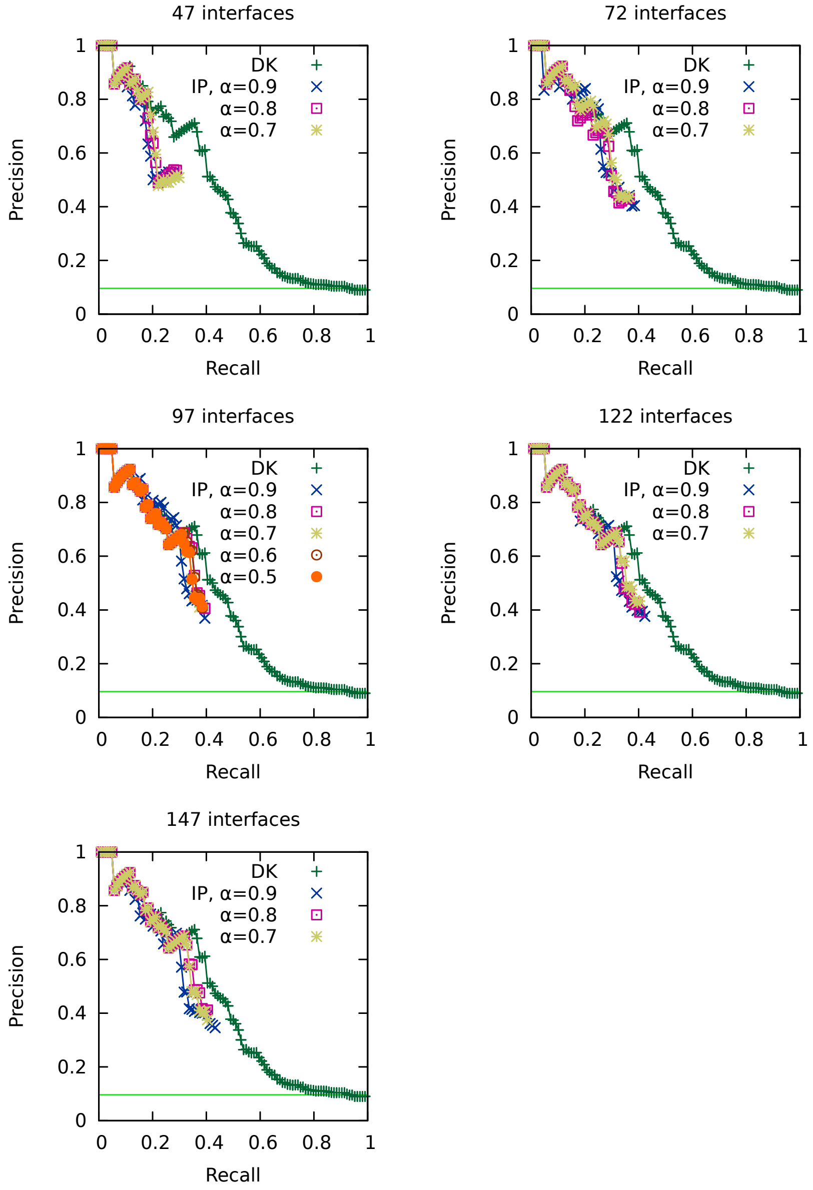

BMV Sign Prediction:
Varying α, the proportion of activating edges
(using literature interactions, cycles disallowed)

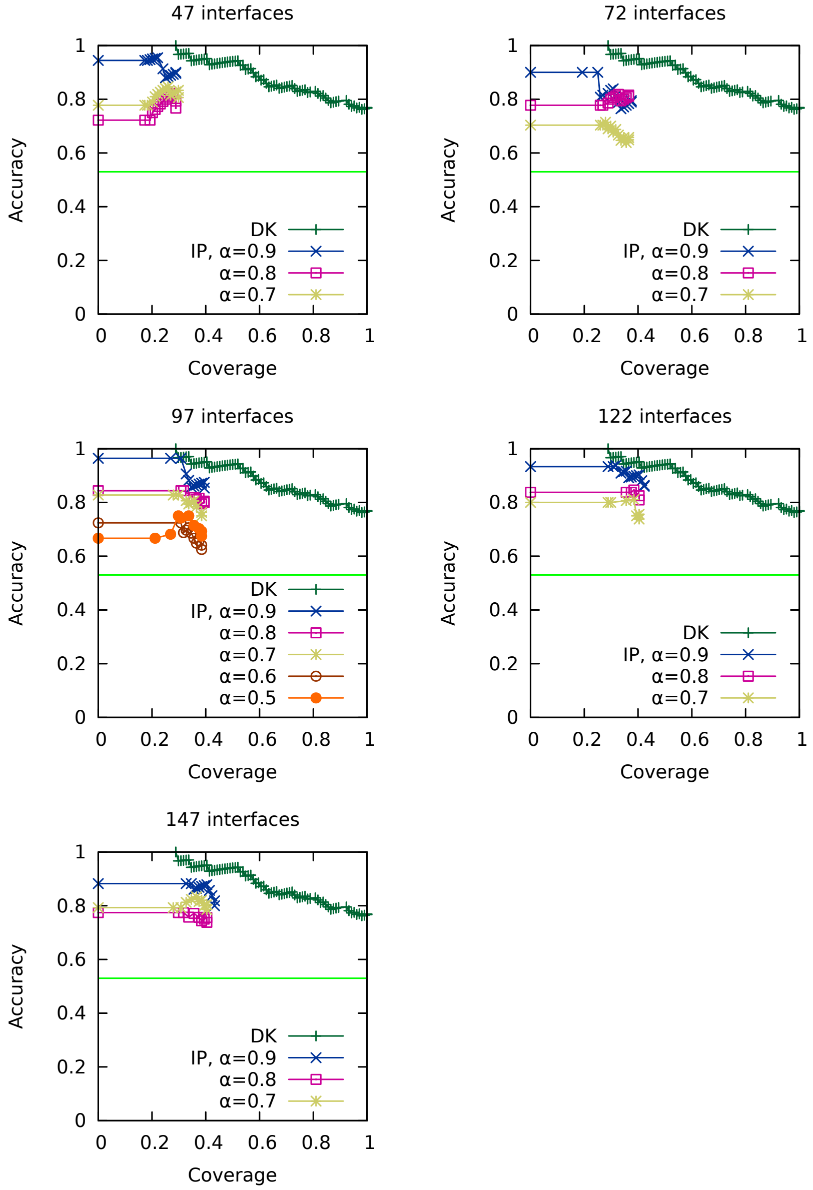

Supplement: Figure S5 — Precision-recall and accuracy-coverage curves showing the effect of varying ; BMV dataset. Results are provided for at all levels of (the number of interfaces). (PDF) [file pcbi.1003626.s005.pdf]
