# Supplementary material for: Inferring Host Gene Subnetworks Involved in Viral Replication
Source: PLoS Comput Biol. 2014 May 29;10(5):e1003626. doi: 10.1371/journal.pcbi.1003626 (PMC4038467; doi:10.1371/journal.pcbi.1003626)
Supplement: Figure S7 — Precision-recall and accuracy-coverage curves assessing accuracy of the cycle-prohibiting constraint; BMV dataset. Results are provided at all levels of (the number of interfaces). (PDF) [file pcbi.1003626.s007.pdf]

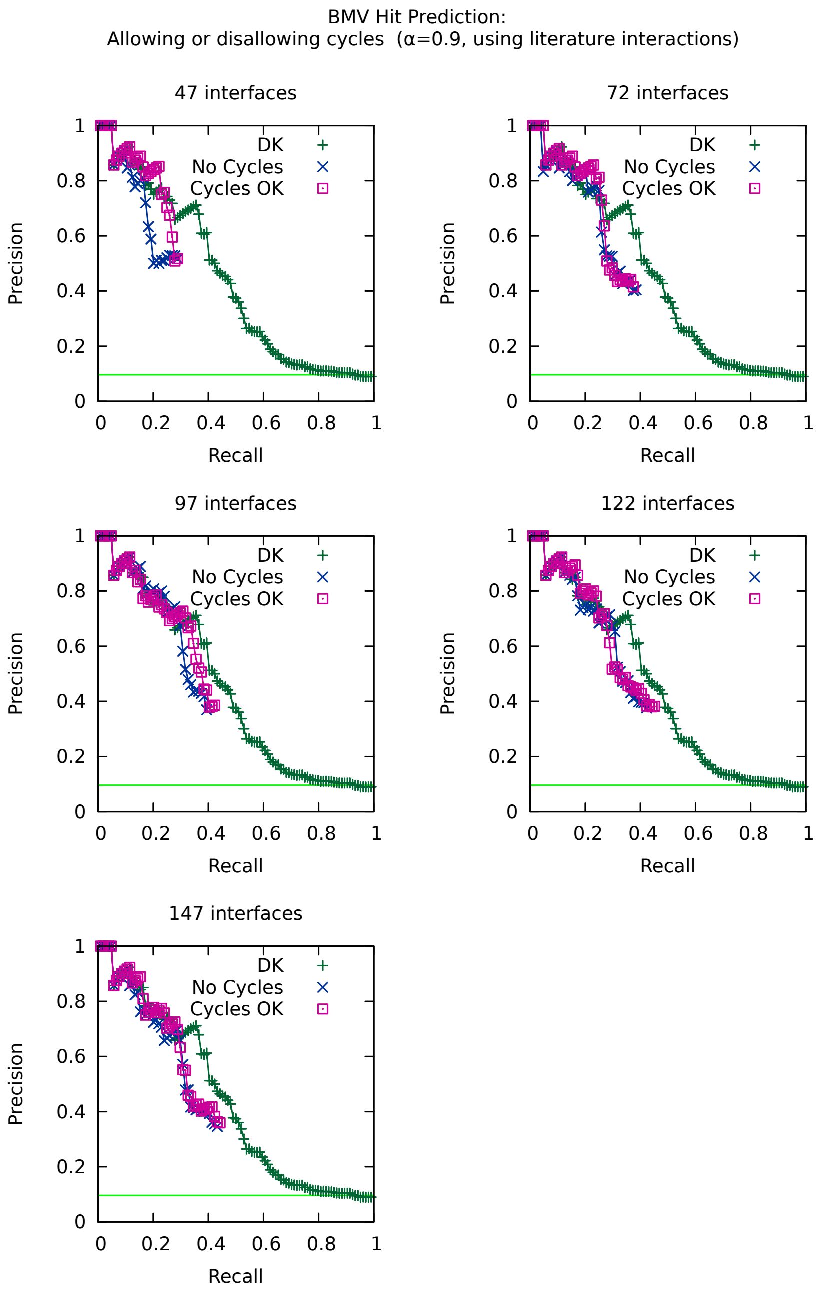
BMV Hit Prediction:
Allowing or disallowing cycles (α=0.9, using literature interactions)
47 interfaces
72 interfaces
97 interfaces
122 interfaces
147 interfaces
Precision
Recall
DK
No Cycles
Cycles OK
0
0.2
0.4
0.6
0.8
1

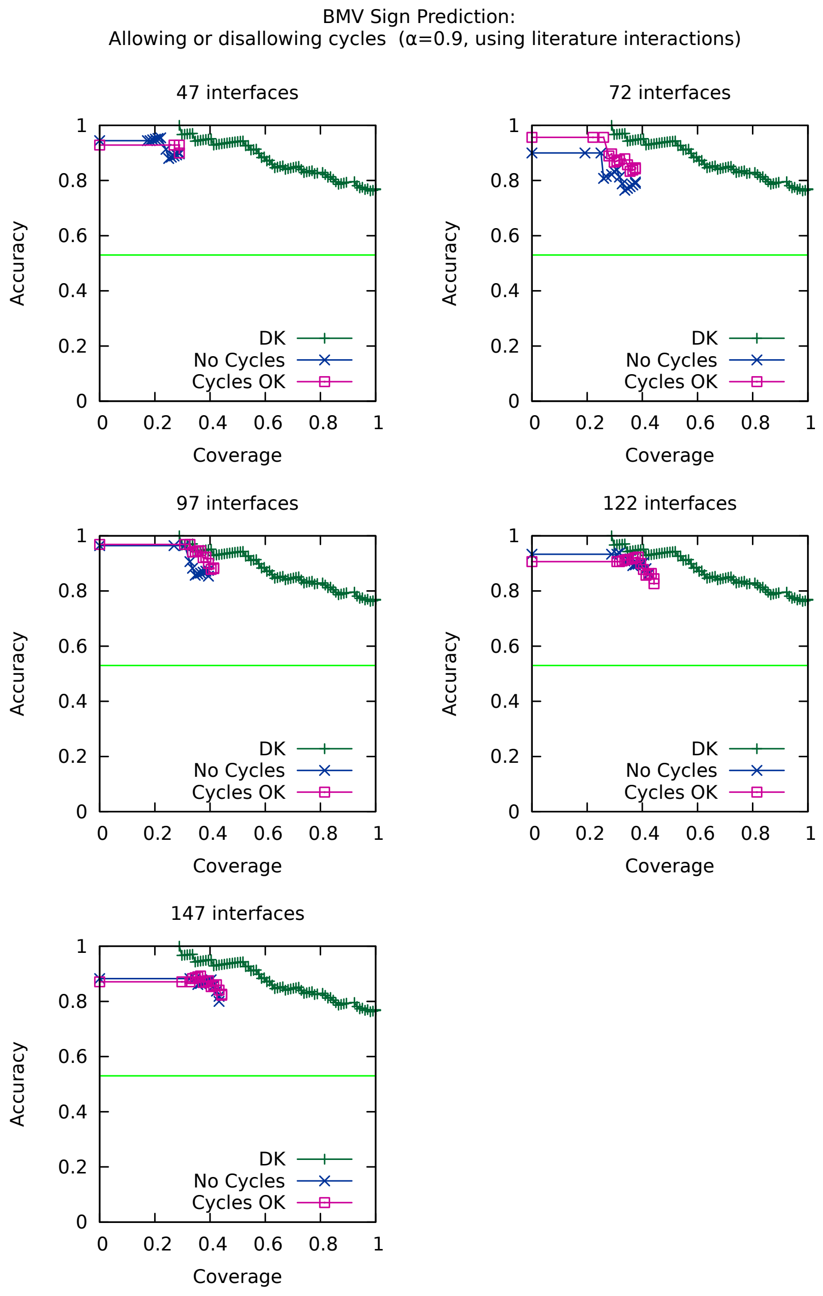
BMV Sign Prediction:
Allowing or disallowing cycles (α=0.9, using literature interactions)
47 interfaces
72 interfaces
97 interfaces
122 interfaces
147 interfaces
Accuracy
Coverage
DK
No Cycles
Cycles OK
0
0.2
0.4
0.6
0.8
1
