# Supplementary material for: Inferring Host Gene Subnetworks Involved in Viral Replication
Source: PLoS Comput Biol. 2014 May 29;10(5):e1003626. doi: 10.1371/journal.pcbi.1003626 (PMC4038467; doi:10.1371/journal.pcbi.1003626)
Supplement: Figure S9 — Precision-recall and accuracy-coverage curves assessing the accuracy of literature-curated interactions. Results are provided for BMV at all levels of (the number of interfaces). (PDF) [file pcbi.1003626.s009.pdf]

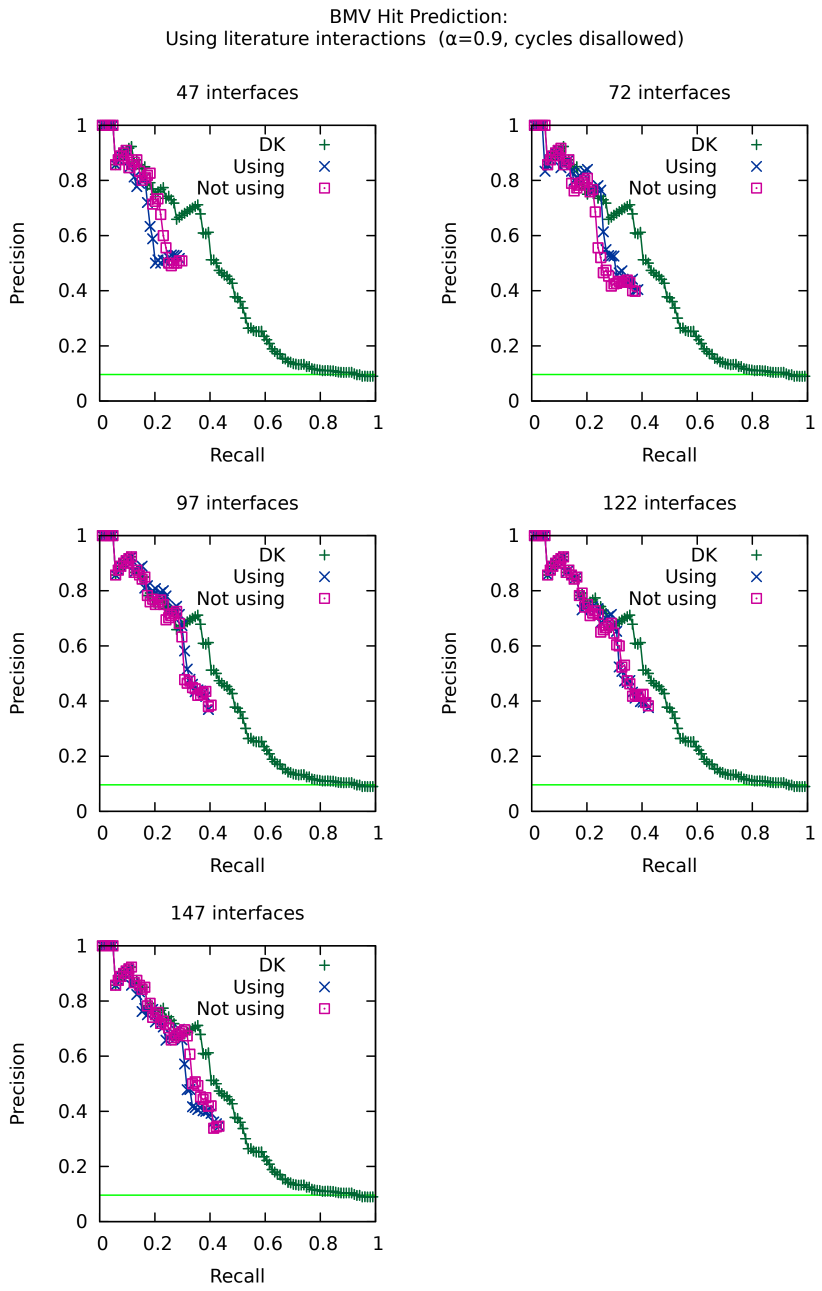
BMV Hit Prediction:
Using literature interactions (α=0.9, cycles disallowed)
47 interfaces
72 interfaces
97 interfaces
122 interfaces
147 interfaces
DK
Using
Not using
Precision
Recall
0
0.2
0.4
0.6
0.8
1

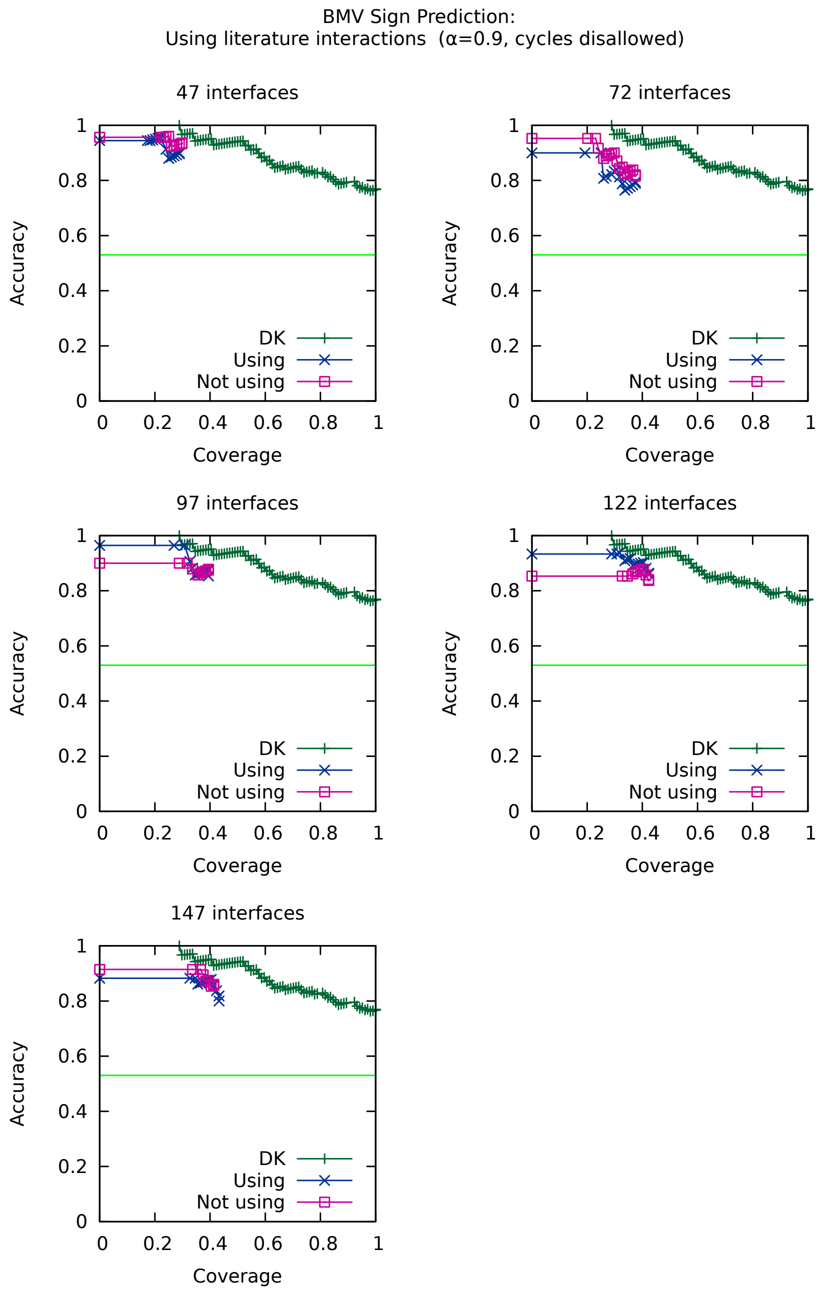
BMV Sign Prediction:
Using literature interactions (α=0.9, cycles disallowed)
47 interfaces
72 interfaces
97 interfaces
122 interfaces
147 interfaces
DK
Using
Not using
Accuracy
Coverage
0
0.2
0.4
0.6
0.8
1
