# Supplementary material for: Inferring Host Gene Subnetworks Involved in Viral Replication
Source: PLoS Comput Biol. 2014 May 29;10(5):e1003626. doi: 10.1371/journal.pcbi.1003626 (PMC4038467; doi:10.1371/journal.pcbi.1003626)
Supplement: Table S1 — Stability of leave-one-out inferred subnetworks. Stability of predictions for all settings of . (PDF) [file pcbi.1003626.s010.pdf]

Table S1: **Stability of leave-one-out inferred subnetworks**

Data set	γ	Node relevance	Node phenotype signs	Interface predictions	Interface relevance
BMV	47	0.853	0.843	0.765	0.878
	72	0.909	0.796	0.734	0.907
	97	0.92	0.803	0.712	0.896
	122	0.928	0.814	0.761	0.897
	147	0.928	0.835	0.776	0.898
FHV	26	0.817	0.796	0.716	0.790
	51	0.891	0.742	0.742	0.867
	76	0.941	0.747	0.82	0.910
	101	0.856	0.795	0.696	0.788
	126	0.889	0.728	0.768	0.847

Stability of leave-one-out ensembles relative to ensembles inferred using all data. For both data sets, we show the stability of four types of predictions across each setting of γ.
