# Supplementary material for: Inferring Host Gene Subnetworks Involved in Viral Replication
Source: PLoS Comput Biol. 2014 May 29;10(5):e1003626. doi: 10.1371/journal.pcbi.1003626 (PMC4038467; doi:10.1371/journal.pcbi.1003626)
Supplement: Table S2 — Sizes of inferred subnetworks. Average counts of weak and unassayed host factors that are predicted to be relevant by the leave-one-out ensembles. (PDF) [file pcbi.1003626.s011.pdf]

Table S2: **Sizes of inferred subnetworks**

Data set	γ	Relevant weak	Relevant unassayed
BMV	47	44.32	112.13
	72	79.96	179.96
	97	107.36	203.56
	122	125.34	219.81
	147	141.50	235.18
FHV	26	20.91	50.10
	51	48.62	100.05
	76	65.84	129.79
	101	80.19	148.86
	126	93.68	171.00

Average number of predicted relevant weak-phenotype and unassayed genes in the leave-one-out inferred subnetworks.
