# Supplementary material for: Inferring Host Gene Subnetworks Involved in Viral Replication
Source: PLoS Comput Biol. 2014 May 29;10(5):e1003626. doi: 10.1371/journal.pcbi.1003626 (PMC4038467; doi:10.1371/journal.pcbi.1003626)
Supplement: Table S3 — Enriched, predicted relevant, protein complexes. List of protein complexes predicted to be relevant and supported by permutation analyses. (PDF) [file pcbi.1003626.s012.pdf]

Table S3: **Enriched, predicted relevant, protein complexes.**

Complex name	Size	Hits	Predicted hits	Permuted subnetworks p-value		Random predictions p-value	
19/22S regulator	22	3	16	< 0.001	*	< 0.001	*
Mediator	25	2	11	0.243		< 0.001	*
20S proteasome	14	2	10	0.007	*	< 0.001	*
Nsp1p complex	4	1	3	0.02	*	0.017	*
nuclear ubiquitin ligase complex	4	1	3	0.04	*	0.021	*
UTP B complex	6	1	3	0.093		0.006	*
THO complex	4	2	2	0.014	*	0.029	*
karyopherin docking subcomplex of the Nuclear Pore Complex	3	1	2	0.048	*	0.014	*
Mot1p complex	2	0	2	0.039	*	0.066	
Decapping Enzyme Complex	2	0	2	0.118		0.018	*
Elongator complex	6	5	1	0.004	*	0.176	
Set3p complex	7	3	1	< 0.001	*	0.378	
UDP-N-acetylglucosamine transferase complex	2	1	1	0.036	*	0.057	
Bub1p/Bub3p complex	2	1	1	0.036	*	0.107	
DSIF complex	2	1	1	0.048	*	0.215	
Lge1p/Bre1p complex	2	1	1	0.002	*	0.034	*
Prs1p/Prs3p	2	1	1	0.05		0.045	*
OCA complex	6	6	0	< 0.001	*	–	
Ski Complex	3	3	0	0.014	*	–	
Nem1p/Spo7p complex	2	2	0	< 0.001	*	–	

Complexes in this table received a p-value of < 0.05 (indicated with *) in either the test based on inferring permuted subnetworks ("Permuted subnetworks"), or the test based on drawing random predicted hits ("Random predictions"). Complexes are listed in decreasing order of the number of predicted hits. The final three rows list complexes that are entirely composed of experimental hits.
