# Supplementary material for: Inferring Host Gene Subnetworks Involved in Viral Replication
Source: PLoS Comput Biol. 2014 May 29;10(5):e1003626. doi: 10.1371/journal.pcbi.1003626 (PMC4038467; doi:10.1371/journal.pcbi.1003626)
Supplement: Table S4 — Interfaces accounted for by enriched complexes. List of high-confidence interfaces that are or are represented by significantly-enriched protein complexes. (PDF) [file pcbi.1003626.s013.pdf]

Table S4: **Interfaces accounted for by enriched complexes.**

Complex name	Interface	Size	Hits	Predicted hits	Permuted subnetworks p-value		Random predictions p-value	
19/22S regulator		22	3	14	0.002	*	< 0.001	*
SCF-Cdc4 complex	Cdc34p	5	1	3	0.015	*	0.017	*
Ada2p/Gcn5p/Ada3 transcription activator complex	Gcn5p	5	0	3	0.027	*	0.051	
Tup1p/Ssn6p complex	Tup1p	2	0	2	0.05		0.049	*
Set3p complex		7	3	1	< 0.001	*	0.174	
UDP-N-acetylglucosamine transferase complex		2	1	1	0.029	*	0.034	*
Nem1p/Spo7p complex		2	2	0	< 0.001	*	–	
OCA complex		6	6	0	< 0.001	*	–	
Ski Complex		3	3	0	0.009	*	–	

Significantly enriched complexes that account for predicted interfaces. If the complex contains an interface and is not a predicted interface itself, the protein name of the predicted interface is listed in the "Interface" column. The final three rows list complexes that are entirely composed of hits.
