# Supplementary material for: Inferring Host Gene Subnetworks Involved in Viral Replication
Source: PLoS Comput Biol. 2014 May 29;10(5):e1003626. doi: 10.1371/journal.pcbi.1003626 (PMC4038467; doi:10.1371/journal.pcbi.1003626)
Supplement: Text S1 — Stability analysis of inferred subnetworks. Analysis of the stability of the predictions made by the cross-validation ensembles. (PDF) [file pcbi.1003626.s014.pdf]

Text S1. Stability analysis of inferred subnetworks

To examine the robustness of our inference method, we measure how well the ensemble inferred using complete experimental data agrees with the ensembles inferred during the leave-one-out experiments. Specifically, we compare four types of predictions: (i) which nodes are relevant (y_n), (ii) the phenotype signs of relevant nodes (v_n when $x_n = 1$), (iii) which nodes are interfaces (x_e for edges from predicted interfaces to the virus), and (iv) the relevance of nodes that are predicted to be interfaces (y_n, considering only nodes that are ever predicted to be interfaces by any ensemble, but regardless of the confidence in that prediction).

We measure the similarity, or agreement, between the predictions of two ensembles E (complete experimental data) and E' (one missing test case) as follows. Using the variable y_n (node relevance) as an example, $p^E(y_n = 1)$ is E's confidence that node n is relevant.

$$similarity(E, E') = 1 - \frac{\sum\limits_{n \in \mathcal{N} - \mathcal{N}^H} |p^E(y_n = 1) - p^{E'}(y_n = 1)|}{\sum\limits_{n \in \mathcal{N} - \mathcal{N}^H} p^E(y_n = 1)}$$

We define stability as the mean similarity between the complete-data ensemble and each leave-one-out ensemble.

Table S1 reports the stability of our method for both BMV and FHV data sets and for each setting of γ. For predictions about node relevance, the ensembles are highly stable, with about half of the values above 0.9. Phenotype sign predictions and predictions about which nodes are interfaces show somewhat lower stability. However, a comparison of the last two columns in the table reveals that predicted interfaces are still likely to be deemed relevant across the set of ensembles, even if they are not as consistently predicted to be interfaces. Overall, predictions for BMV are more stable than predictions for FHV, which may be due to the greater connectivity of BMV's hits compared to FHV's.

In Table S2, we also provide the average number of predicted hits in the ensembles inferred using all experimental hits, separately reporting the number of predicted hits with weak phenotype labels (the "Relevant weak" column) and the number of unassayed predicted hits ("Relevant unassayed").
