# Supplementary material for: Inferring Host Gene Subnetworks Involved in Viral Replication
Source: PLoS Comput Biol. 2014 May 29;10(5):e1003626. doi: 10.1371/journal.pcbi.1003626 (PMC4038467; doi:10.1371/journal.pcbi.1003626)
Supplement: Text S2 — SPINE edge-sign prediction heuristic. Description of the IP variables and constraints used to implement the SPINE-based edge sign prediction heuristic. (PDF) [file pcbi.1003626.s015.pdf]

Text S2. SPINE edge-sign prediction heuristic

In their SPINE method for inferring signaling networks from source-target pairs, Ourfali *et al* [1] employ the assumption that each node is either a repressor or an activator: that is, all edges leaving from a node must have the same sign. We have adapted this heuristic for our integer program in order to compare it to our own heuristic, which simply requires that, globally, at least 90% of edges must be activating. This is achieved with the introduction of two new variables per node and four new constraints per edge (and fewer for edges that have a fixed sign).

We introduce two new binary variables: a_n, which is set to 1 if the node is predicted to be an activator, and h_n, which is set to 1 if the node is predicted to be a repressor (in*h*ibitor). Every node is constrained such that it can only ever be one of the two:

$$\forall\ n \in \mathcal{N} \quad y_n = a_n + h_n$$

If a node n is an activator, then any relevant outgoing edge e must be activating; likewise, if n is an inhibitor, so are all of its outgoing edges. For n's outgoing directed edges, this is fairly straightforward to constrain. (The set $\mathcal{E}^I - \mathcal{E}^U$ refers to directed edges.)

$$\begin{aligned}\forall e = (n_i, n_j) \in \mathcal{E}^I - \mathcal{E}^U \quad & a_e \leq a_{n_i} \\ & h_e \leq h_{n_i}\end{aligned}$$

For undirected edges, however, an edge's sign is only tied to a node's activator/repressor status if the node is the source of the edge. $out(e, n)$ specifies the value of d_e for which n is the source of the edge.

$$\begin{aligned}\forall e = (n_i, n_j) \in \mathcal{E}^U \quad & a_e + out(e, n_i) \leq 1 + a_{n_i} \\ & h_e + I(d_e = out(e, n_i)) \leq 1 + h_{n_i}\end{aligned}$$

References

1. Ourfali O, Shlomi T, Ideker T, Ruppin E, Sharan R (2007) SPINE: a framework for signaling-regulatory pathway inference from cause-effect experiments. Bioinformatics 23: i359–i366.
