# Supplementary material for: Inferring Host Gene Subnetworks Involved in Viral Replication
Source: PLoS Comput Biol. 2014 May 29;10(5):e1003626. doi: 10.1371/journal.pcbi.1003626 (PMC4038467; doi:10.1371/journal.pcbi.1003626)
Supplement: Text S3 — Permutation analysis of inferred relevant complexes. Further analysis of predicted relevant protein complexes. (PDF) [file pcbi.1003626.s016.pdf]

Text S3. Permutation analysis of inferred relevant complexes

We perform two Monte Carlo analyses to assess the representation of complexes by our inferred subnetworks. We separately consider inferred relevant complexes and complexes that are or contain high-confidence interfaces.

Representation of complexes among experimental and predicted hits

When we considered the biological plausibility of predicted-but-unassayed host factors for BMV, we noted that a number of them are members of large protein complexes, including the proteasome and Mediator. The 97-interface inferred BMV subnetwork contains 65 protein complex nodes with confidence ≥ 0.75.

In a first permutation test, we assess whether the degree to which protein complexes are represented in the inferred subnetwork is simply due to properties of the underlying background network, independent of the experimental data. First, for each of the 65 complexes that are predicted to be relevant, we count how many of its constituent genes are experimental or predicted hits. Next, we estimate a distribution of random inferred subnetworks by applying a 0.75-confidence threshold to each of the 1,000 ensembles that we inferred from permuted data to generate baselines for the hit- and sign-prediction tests. For each complex, we record the fraction of the complex that is represented by permuted and predicted hits in each random subnetwork. Finally, we calculate a permutation-based p-value for each complex as the proportion of permutations that contain as many or more members of the complex.

Out of 65 inferred relevant complexes, 16 are better represented (at $p < 0.05$) by our inferred BMV subnetwork than by random inferred subnetworks. This result demonstrates that the representation of many of the complexes in our inferred subnetworks is not merely due to the topology of the background network. Among these 16 are two components of the proteasome: the 20S proteasome and its 19/22S regulatory particle. However, the Mediator complex, another large complex predicted to be relevant by our method, is not significantly better represented by our inferred subnetwork than by random subnetworks. While we cannot rule out the possibility that our method inferred it to be relevant simply because it is highly central in the background network, it is still possible that the Mediator is actually relevant to viral replication.

In a second Monte Carlo test, we consider whether the degree of involvement of *predicted* hits in relevant complexes is due to properties of the underlying background network and experimental data, independent of the *inference procedure*. For this test, we measure the representation of each complex by the set of predicted hits from the inferred BMV subnetwork. To estimate a random distribution of predicted hit sets, we draw 1,000 random subsets of the weak-phenotype genes and unassayed genes, in the same size and degree distribution as the set of predicted hits from the inferred BMV subnetwork. Again, we calculate the permutation-based p-value for a complex as the fraction of random predicted hit sets that contain as many or more members of the complex.

Eleven out of the 65 predicted relevant complexes are better represented ($p < 0.05$) by the inferred BMV subnetwork than by random hit predictions, including the two proteasome complexes as well as the Mediator. This result shows that our method can predict the inclusion of complexes that would not have been just as well-represented by randomly drawn genes.

Table S3 shows the predicted relevant complexes that are significant under either of these tests.

Representation of complexes that are used as interfaces

We use another pair of analogous Monte Carlo tests to examine the high-confidence, predicted BMV-yeast interfaces that are protein complexes or are members of complexes. Six high-confidence interfaces are themselves complexes, three of which are entirely composed of hits and are not considered during this analysis. Another five are genes are members of one or two complexes in the background network.

For the first test, we assess the representation of the interface-related complexes in 1,000 subnetworks inferred using permuted data and a minimum number of interfaces (the median was 52). Five out of the eight interfaces that are related to complexes that are better represented by the inferred BMV subnetwork than by random subnetworks ($p < 0.05$), including all three of the complexes that are used directly as interfaces.

For the second test, we assess the representation of the interface-related complexes by randomly drawn subsets of weak and unassayed genes. Four out of the eight complexes are significantly better represented by the inferred BMV subnetwork than by a random gene set ($p < 0.05$), including two out of the three complexes that are directly used as interfaces.

Taken together, the results of both of these analyses indicate that the complexes that our method predicts to be interfaces are well-supported by predicted hits and are not likely to be artifacts of the background network or chance. Table S4 shows the predicted relevant interfaces that can be accounted for by a complex that has a significant p-value in either of the Monte Carlo tests.
